# Genome-Wide mRNA Expression Analysis of Hepatic Adaptation to High-Fat Diets Reveals Switch from an Inflammatory to Steatotic Transcriptional Program

**DOI:** 10.1371/journal.pone.0006646

**Published:** 2009-08-14

**Authors:** Marijana Radonjic, Jorn R. de Haan, Marjan J. van Erk, Ko Willems van Dijk, Sjoerd A. A. van den Berg, Philip J. de Groot, Michael Müller, Ben van Ommen

**Affiliations:** 1 Nutrigenomics Consortium, Top Institute Food and Nutrition, Wageningen, The Netherlands; 2 TNO Quality of Life, BU Biosciences, Zeist, The Netherlands; 3 Departments of General Internal Medicine and Human Genetics, Leiden University Medical Center, Leiden, The Netherlands; 4 Department of Human Nutrition, Wageningen UR, Wageningen, The Netherlands; Institute of Preventive Medicine, Denmark

## Abstract

**Background:**

Excessive exposure to dietary fats is an important factor in the initiation of obesity and metabolic syndrome associated pathologies. The cellular processes associated with the onset and progression of diet-induced metabolic syndrome are insufficiently understood.

**Principal Findings:**

To identify the mechanisms underlying the pathological changes associated with short and long-term exposure to excess dietary fat, hepatic gene expression of ApoE3Leiden mice fed chow and two types of high-fat (HF) diets was monitored using microarrays during a 16-week period. A functional characterization of 1663 HF-responsive genes reveals perturbations in lipid, cholesterol and oxidative metabolism, immune and inflammatory responses and stress-related pathways. The major changes in gene expression take place during the early (day 3) and late (week 12) phases of HF feeding. This is also associated with characteristic opposite regulation of many HF-affected pathways between these two phases. The most prominent switch occurs in the expression of inflammatory/immune pathways (early activation, late repression) and lipogenic/adipogenic pathways (early repression, late activation). Transcriptional network analysis identifies NF-κB, NEMO, Akt, PPARγ and SREBP1 as the key controllers of these processes and suggests that direct regulatory interactions between these factors may govern the transition from early (stressed, inflammatory) to late (pathological, steatotic) hepatic adaptation to HF feeding. This transition observed by hepatic gene expression analysis is confirmed by expression of inflammatory proteins in plasma and the late increase in hepatic triglyceride content. In addition, the genes most predictive of fat accumulation in liver during 16-week high-fat feeding period are uncovered by regression analysis of hepatic gene expression and triglyceride levels.

**Conclusions:**

The transition from an inflammatory to a steatotic transcriptional program, possibly driven by the reciprocal activation of NF-κB and PPARγ regulators, emerges as the principal signature of the hepatic adaptation to excess dietary fat. These findings may be of essential interest for devising new strategies aiming to prevent the progression of high-fat diet induced pathologies.

## Introduction

Pathologies associated with metabolic syndrome, such as overweight and obesity, insulin resistance, hypertension, hyperlipidemia, non-alcoholic hepatic steatosis and diabetes are becoming a health problem of epidemic proportions in Western societies [1]–[3]. In addition to genetic factors, diet represents the most important determinant of the development and progression of the metabolic syndrome [4]. High-fat diets have been shown to induce obesity and insulin resistance in humans and rodents [5]–[7]. It is believed that short-term high-fat feeding triggers a stress response (termed “metabolic stress”), which challenges the system to adapt to the new conditions and maintain homeostasis. If the fat overload persists, the system fails to adjust and consequently undergoes pathophysiological changes characteristic for metabolic syndrome [8], [9]. The early diagnostics of metabolic stress and the elucidation of the molecular mechanisms underlying a transition from the early to late stages of metabolic syndrome are of crucial importance for developing intervention strategies for preventing irreversible disease characteristics.

It is generally acknowledged that excessive exposure to dietary lipids disrupts the homeostasis of cellular metabolism and triggers an inflammatory response in adipose tissue [10]. Nevertheless, the dynamics and coordinate regulation of processes perturbed by excess dietary fat in other organs is scarcely understood. Several recent studies highlighted the important role of lipid-activated nuclear receptors such as peroxisome-proliferator-activated receptors (PPARs) in the integration of metabolic and inflammatory processes [11]. In addition to their role as transcription activators of metabolic genes, PPARs are receiving an increasing attention as inhibitors of inflammatory gene expression achieved primarily through suppression of the pro-inflammatory NF-κB pathway [11], [12]. This dual function makes PPARs attractive targets for intervention in both metabolic and inflammatory disorders, although little is known about role of PPARs in the diseases where the cross-talk of these pathways may be fundamental to the development of pathogenesis [13]–[15]. Despite the significant progress in the field, much work is still required to fully understand the molecular mechanisms underlying the effects of high-fat diets and delineate their coordination during onset and progression of metabolic syndrome on organ and systems level.

In this study, we have used Apolipoprotein E3-Leiden (ApoE3L) mice to investigate the effect of two types of standard laboratory high-fat (HF) diets on development of metabolic syndrome. ApoE3L transgenic mice are an established model for studying the effect of dietary interventions on hyperlipidemia, atherosclerosis and other diet-related pathologies [16]–[23]. Due to the expression of the human APOE*3Leiden and apoC1 gene cluster in C57BL/6J (B6) background, the ApoE3L mice display a lipoprotein profile that closely resembles that of humans, and they develop human-like dysbetalipoproteinemia and atherosclerotic lesions when fed Western-type diets [24], [25]. We show that ApoE3L mice fed either a beef tallow- or palm oil- based high-fat diet (HFBT and HFP, respectively) for 16 weeks develop metabolic syndrome characteristics, such as obesity and hepatic steatosis. By monitoring the genome-wide hepatic mRNA expression of these mice at eight time-points, covering the period from the beginning of the high-fat feeding until the occurrence of significant changes in metabolic syndrome parameters (week 16), we could construct a comprehensive view of the biological processes characteristic of hepatic adaptation to excess dietary fat during the progression from metabolic stress to metabolic syndrome. The reciprocal activation of the inflammatory/immune response and the lipogenic/adipogenic pathways emerges as the most prominent signature of the transition from short to long-term HF feeding and underscores the relevance of the antagonistic action of NF-κB and PPARγ regulators in controlling the shift from the stressed, inflamed to the pathological, steatotic hepatic state. These results provide novel insights into the interaction between metabolic and inflammatory processes during the development of metabolic syndrome that may be important when considering strategies to prevent and treat the disease.

## Results

**Figure 1:**
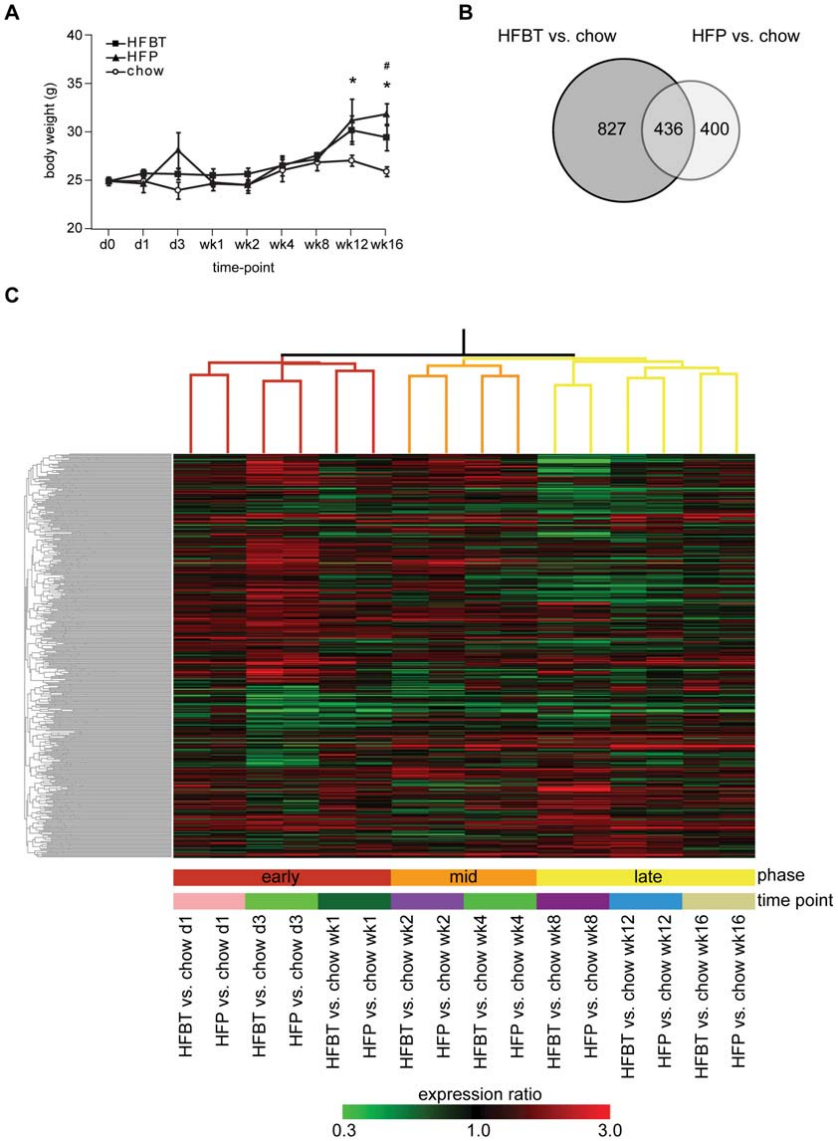
Increased body weight and gene expression changes induced by HFBT and HFP high-fat diets. (A) Average body weight of ApoE3L mice fed either chow, HFBT or HFP diet in each time point of the 16-week time course. Error bars represent standard deviation within a group. Statistically significant (p<0.05) increase in body weight of HFBT and HFP fed mice compared to chow fed mice are marked with asterisk and hash sign, respectively. (B) Overlap between the total numbers of statistically significant differentially expressed genes in livers of ApoE3L mice fed either HFBT or HFP diet compared to chow diet per time-point, over the 16-week time-course. (C) Hierarchical clustering (Pearson correlation, complete linkage) of 16 experimental conditions (two high-fat diets at 8 time-points) and 1663 genes differentially expressed under either of two high-fat conditions. Values used for clustering are average HFBT vs. chow and HFP vs. chow per time-point expression ratios. The branches of the condition tree are colored so to discriminate three subclusters with the largest distance, corresponding to three phases of the time-course: early (red), mid (orange) and late (yellow). This is summarized in the color bar underneath the cluster diagram. The lower color bar indicates distinct time-points, stressing the similarity of HFBT and HFP transcriptional response at each point of the time-course.

To investigate the processes associated with the high-fat (HF) induced metabolic stress and the progression of the metabolic syndrome, male ApoE3L mice were fed one of the three standard laboratory diets: chow (control diet), HFBT (45 energy %, beef tallow high-fat diet containing 0.25% cholesterol) and HFP (45 energy % palm oil high-fat diet), for a period of 16 weeks (Materials and methods). The primary aim of the study was to identify the effects of excessive dietary fat content and to determine how these effects change over time. The additional aspect that we aimed to address was to which extent parameters other than percentage of fat determine the consequences of HF feeding. Dietary parameters, such as fat origin or presence or absence of cholesterol, may also be relevant determinants of HF diet effects. To address this aspect, cholesterol-containing animal fat-based diet (HFBT, also referred to as Western-type diet) and plant oil-based diet with relatively high amount of saturated fats (HFP) were investigated in parallel. During the 16 week experimental period the body weight of both HFBT and HFP fed mice increased significantly compared to the chow-fed mice (Figure 1A).

To focus on the molecular mechanisms underlying the development of metabolic syndrome, hepatic mRNA expression of HF- and chow-fed ApoE3L mice (n = 150) was monitored using DNA microarrays over a period of 16 weeks. At the day 0 and eight additional time-points (day 1, day 3, week 1, 2, 4, 8, 12 and 16) mice were sacrificed, their livers were sampled and the RNA expression was analysed using NuGO Affymetrix mouse arrays [26]. After the quality control and the preprocessing of data, expression values were obtained for 15105 genes in 3 to 6 biological replicate samples per diet and time-point (Materials and methods).

### Global temporal changes in hepatic transcriptome during the 16-week time-course

To assess temporal changes in hepatic gene expression over the 16-week period under the control and two high-fat diets, each of the time points per diet was compared to time-point day 0 in a pairwise fashion using limma statistical package [27]. Applying statistical cutoff of false discovery rate (FDR) <0.1, we identified 839, 3027 and 3316 genes differentially expressed by chow, HFBT and HFP feeding, respectively, across any of eight time-points (Figure S1). This showed that ageing of animals from 14 weeks (at the beginning of the experiment) to 30 weeks (at its end) affected expression of a portion of genes, also in the control condition. Nevertheless, the combined effects of ageing and high-fat feeding observed in HFBT and HFP conditions was substantially larger compared to solely aging effects.

In addition to overall temporal effects, comparing each of the time points to day 0 allowed assessment of dynamics of transcriptional response by detecting the magnitude of the gene expression changes at each time point compared to the starting condition. In both HF conditions we observed three phases of hepatic transcriptional response characterized by local peaks in the number of differentially expressed genes: early (day 1 to week 1), mid (week 2 and week 4) and late (week 8 to week 16) (Figure S1). In addition to the phasic trend, the number of differentially expressed genes in HF conditions increased gradually during the course of the experiment. This may be expected considering that using day 0 as a common reference leads to combined contribution of both high-fat intervention and ageing to the observed effects. In chow condition, temporal trends identified for HF diets were absent and the numbers of changed genes in each of the time-points remained below the ones identified in HF conditions (Figure S1).

### Hepatic transcription response to high-fat diets

After determination of the temporal effects of all three diets across 16-week time-course, we focused on dissecting the HF-specific effects on the hepatic transcriptome. To analyse the effects of HF diets independently of changes occurring due to the animal age, we compared the gene expression of mice fed HFBT and HFP diets to those of mice fed the chow diet in each of the corresponding time-points. The pairwise comparisons were performed using the limma statistical package, and FDR q-value<0.1 was used as a threshold for significance. The numbers of identified differentially expressed genes in each diet and time-point are shown in Table 1 and Table S1. Per time-point, the number of up- and down-reglated genes was largely balanced, and genes changed expression in the same direction in both HF conditions (Table S1). In total, during the 16-week time-course the HFBT diet significantly affected the expression of 1263 genes, while HFP diet affected 836 genes (8.3% and 5.5% of the monitored genome, respectively). The statistically significant differentially expressed genes (DEG) under the two HF diets largely overlap (436 genes, p-value 1.7E-265) (Figure 1B).

**Table 1:** Number of statistically significant differentially expressed genes per time-point (FDR q-value<0.1).

Time point	HFBT vs. chow	HFP vs. chow	Overlap (HFBT vs. chow) and (HFP vs. chow)
Day 1	0	0	0
Day 3	550	416	193
Week 1	88	82	31
Week 2	317	136	76
Week 4	33	44	9
Week 8	7	209	2
Week 12	521	144	85
Week 16	23	52	7
All time-points	1263	836	436
Total (both diets, all time-points)			1663

To compare gene expression patterns of identified HFBT and HFP DEGs across all conditions, expression profiles of genes that are changing in either of HF diets compared to chow diet per time-point (1663 genes in total) were hierarchically clustered (Figure 1C). The close proximity of condition tree branches corresponding to two diets in each of the time-points revealed a striking similarity between the effects of HFBT and HFP diets on the expression of all 1663 genes. Therefore, not only 436 overlapping genes, but also the remaining genes unique to each condition changed similarly under the HFBT and HFP diets, although they did not pass significance threshold in both conditions. Due to this highly comparable gene expression response, the union of differentially expressed genes under the HFBT and HFP diets was further considered as a total set of 1663 HF-responsive genes (Table S1, Table S2). This set of HF-responsive genes was used for all downstream functional analyses that required a limited set of genes of interest as input. Nevertheless, to be able to assess subtle differences between the effects of two HF diets caused by different fat origin and/or the presence or absence of cholesterol in a diet, gene expression changes are always displayed separately for each of two diets.

Three distinct phases (early, mid and late) of hepatic transcriptional response to HF diets were observed by comparing each time-point to day 0 (Figure S1). These phases are also revealed by hierarchical clustering of the experimental conditions in Figure 1C, where gene expression of mice fed HFBT and HFP diets was compared to those of mice fed the chow diet at each of the corresponding time-points. To further characterize the expression profiles of the 1663 HF-responsive genes, Smoothing Spline Clustering was performed [28] (Figure S2, S3, S4 and S5). The identified gene expression profiles typically show peaks at early (day 3), mid (week 2) and/or late (week 8/12) phase of the time-course. Especially prominent expression changes were observed at the early and the late phase, as determined by the intensity of the gene expression changes and the abundance of the significant differentially expressed genes (Table 1, Figure 1C, Table S1, Table S2). Interestingly, many genes change the direction of their regulation between the early and the late phase of the time-course (Figure 1C, Figure S2, Figure S3, Figure S4 and Figure S5). Similarly to the observation obtained by comparing each time-point to day 0, the peak-intervening time-points are characterized by a more modest transcription response and may reflect resumptions of the local homeostasis resulting from the transient adaptation to excess dietary fat.

**Figure 2:**
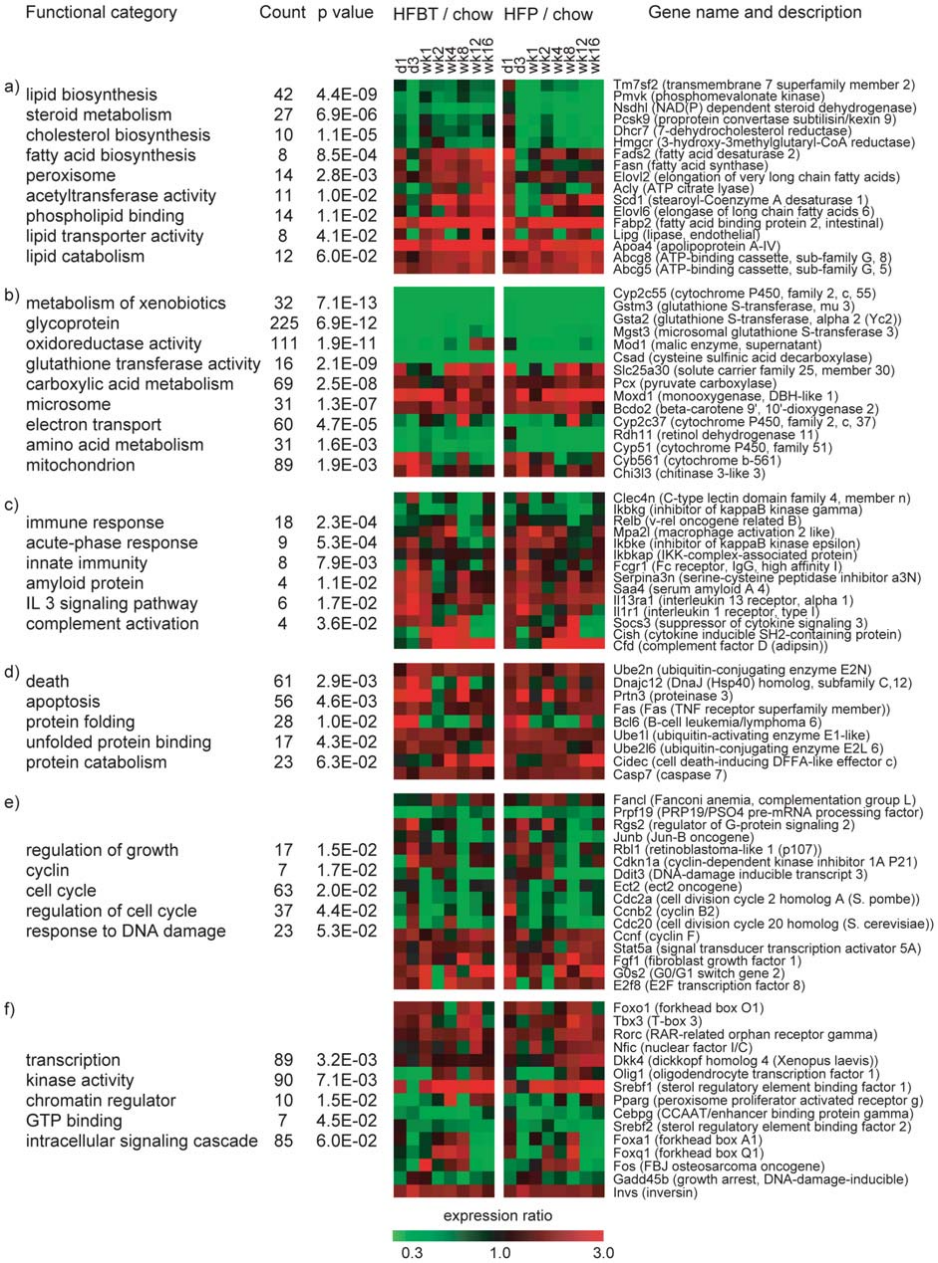
Functional characterization of the high-fat responsive genes. Representative overrepresented functional categories in the set of 1663 high-fat responsive genes are grouped according to their biological function: (a) lipid and cholesterol metabolism, (b) oxidative and metabolic processes, (c) inflammatory and immune response, (d) apoptosis and protein folding, (e) cell growth and cell cycle and (f) transcription regulation and signal transduction. For each functional group, representative genes are listed and their expression profiles (average HFBT vs. chow and HFP vs. chow expression ratios per time-point) are shown in the adjacent diagrams.

To identify which cellular processes are most affected by the hepatic exposure to excess dietary fat over the entire time-course, we first analyzed the overrepresented functional categories among the 1663 HF-responsive genes [29]. The most prominent significantly enriched functional clusters are related to lipid, cholesterol and oxido-reductive metabolism, as well as inflammation, immune response, apoptosis, cell cycle, protein folding and the regulatory pathways controlling these processes (Figure 2, Table S3 (“All HF-responsive genes”)). The expression changes per diet and time-point of the selected representative genes for each functional cluster are also shown in Figure 2. To assess if some of the categories are specifically enriched in either up- or down-regulated genes, we additionally performed the equivalent type of analysis using separately lists of HF-responsive up- and downregulated genes (Table S3 (“Upregulated HF-responsive genes” and “Downregulated HF-responsive genes”)). As a number of genes changed the direction of their regulation between the early and the late phase of the time-course, many categories were represented in both lists. Specifically, the enrichment of lipid metabolism and inflammatory processes were highly significant among the upregulated HF-responsive genes, and glutathione metabolism and cholesterol biosynthesis were most significant among the downregulated HF-responsive genes.

### Integrative temporal and functional characterization of the hepatic transcription response to high-fat diets

**Figure 3:**
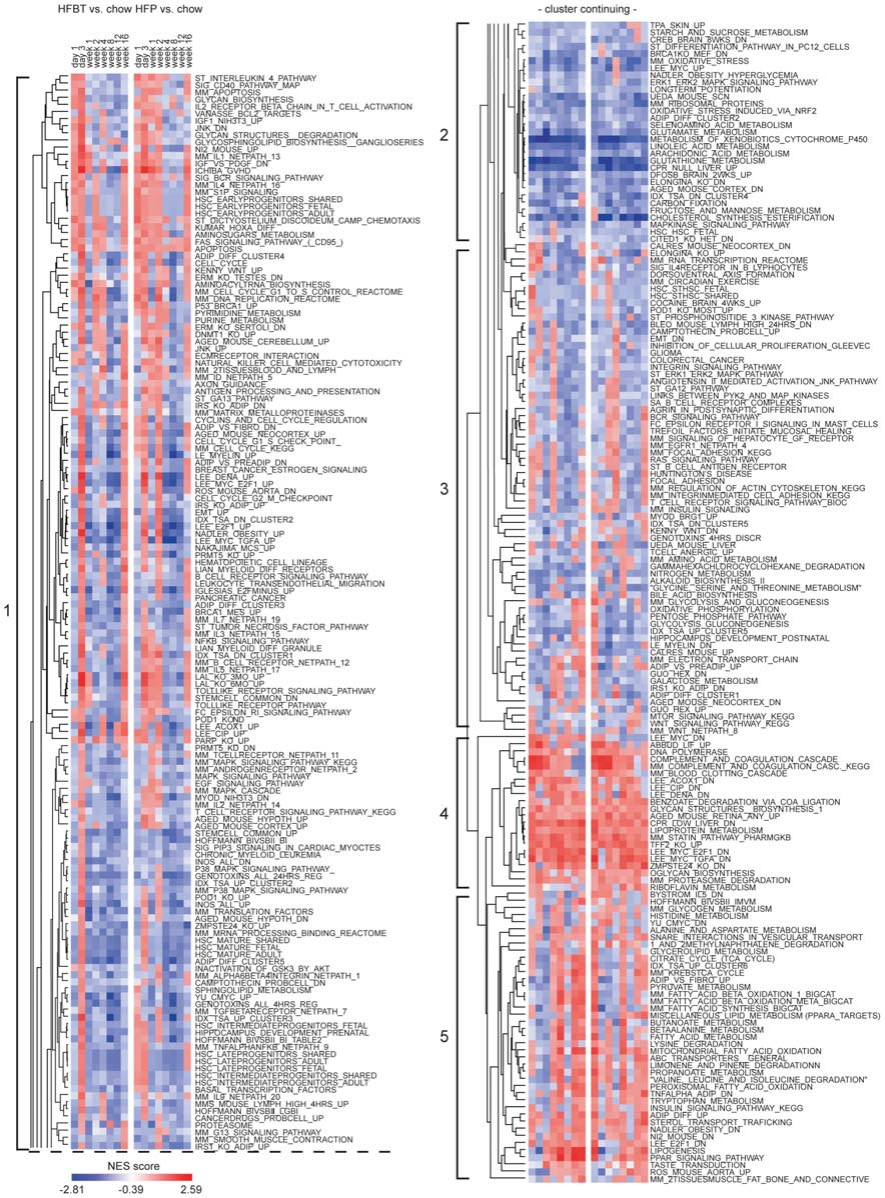
Temporal modules of pathway activities during the hepatic high-fat response. Hierarchical clustering (Pearson correlation (uncentered), average linkage) of Normalized Enrichment Scores (NES), as determined by Gene Set Enrichment Analysis. The NES values of 314 gene sets, significant (FDR q-value<0.1) in at least one of HFBT vs. chow and HFP vs. chow per time-point comparisons are used as an input for hierarchical clustering. The NES scores, represented by the color gradient, correspond to the relative up- (red) and down- (blue) regulation of the gene sets under each of experimental conditions. The cluster diagram can be divided into five main temporal themes (depicted as modules 1 to 5), highlighting the main trends in temporal pathway activities: (1) early activation/late repression; (2) constant repression; (4) constant activation and (5) early repression/mid and late activation. Module (3) includes fuzzy pathway profiles that bring to light differences in transcription response to beef tallow- (HFBT) and palm oil-based (HFP) high-fat diets.

To include prior biological knowledge in pathway analysis, both public and proprietary gene sets were used for Gene Set Enrichment Analysis (GSEA) [30] of each of the HFBT and HFP versus chow per time-point comparisons. In addition, to facilitate the dynamic interpretation of the identified biological functions, statistically significant GSEA results were hierarchically clustered across all conditions, thus integrating temporal and functional information into single visual output (Materials and methods). Hierarchical clustering of the GSEA-calculated normalized enrichment scores (NES) resulted in an aggregation of the similarly regulated gene sets, facilitating the visualization of pathway activities during the 16-week response to HF diets. The resulting cluster of 314 gene sets, significant (FDR q-value<0.1) in at least one of the HF conditions, can be visually divided into five temporal modules (Figure 3).

An examination of the cluster heatmap reveals that the vast majority of the gene sets change the direction of their transcriptional regulation throughout the time-course. The first and largest module contains gene sets that are generally significantly upregulated during the early phase (day 3) and downregulated at the late phase of the HF-response (week 8, 12 and/or 16). The most characteristic gene sets in the module 1 are associated with inflammation and immune response and their regulation, such as Interleukin (IL)-1, IL-2, IL-3, IL-4, IL-5, IL-7 and IL-9 pathways, CD40 pathway, antigen processing and presentation, T and B cell receptors signaling, natural killer (NK) cell cytotoxicity, leukocyte migration and Tumor necrosis factor (TNFalpha), Nuclear factor-kappa B (NF-κB) and Toll-like Receptor (TLR) signaling pathways. The second representative functional theme in module 1 is related to cell growth, proliferation and differentiation. Examples of these gene-sets include cyclins and cell cycle regulators, G1 to S and G2 to M checkpoint controllers, DNA replication reactome and Mitogen-activated protein (MAP) kinases, Epidermal growth factor (EGF) and Transforming growth factor beta (TGF-β) signaling pathways. Additionally, various gene sets related to cancer and development also follow the early induction/late repression expression pattern.

In contrast to module 1, gene sets in the last module (5) are generally repressed during the early phase and significantly upregulated during mid and late phases. The most prominent functional characteristic of module 5 is the presence of the PPAR signaling pathway as well as many PPARs-regulated gene sets, including those associated with adipocytes differentiation (IDX_TSA_UP_CLUSTER6, ADIP_VS_FIBRO_UP, ADIP_DIFF_UP, NADLER_OBESITY_DN), fatty acid oxidation and lipogenesis. The transcriptional activation of these gene sets during the mid phase and their amplification during the late phase implies an important role for PPARs in regulating the transition from short- to long-term effects of hepatic exposure to excess dietary fat. Additionally, hepatic activation of the genes involved in adipocytes differentiation and lipogenesis suggests that fat accumulation and adipogenic transformation likely take place in the liver after long-term exposure to a high-fat diet. Other aspects of the metabolic control, such as amino acid metabolism and tricarboxylic acid cycle, are also upregulated during mid and late phases of the HF-response.

Despite the large overall similarity in transcription response to HFBT and HFP diets, a comparison of pathway activities reveals specific differences between the two high-fat conditions. This is particularly evident in the gene sets clustered in the lowest part of the module 3. Notably, the regulation of gene sets involved in energy metabolism (glycolysis and gluconeogenesis, oxidative phosphorylation, pentose phosphate pathway, electron transport chain) is sensitive to variations in fat origin and/or specific compositions of HF diets. Specifically, a palm oil-based HF diet (HFP) causes a transient induction of the gene sets involved in energy metabolism at day 1 but shows attenuated induction of these gene sets in the late phase compared to a beef tallow-based HF diet (HFBT). Similar deviation in the gene set activity patterns between HFP and HFBT conditions is also visible in the module 5.

Finally, a small fraction of all the represented gene sets retains a constant transcriptional pattern throughout the time-course. These are represented in modules 2 (constantly downregulated gene sets, e.g. cholesterol biosynthesis, glutathione metabolism and metabolism of xenobiotics by cytochrome P450) and module 4 (constantly upregulated gene sets, such as lipoprotein metabolism).

The reciprocal transcriptional profiles of the pathways represented in modules 1 and 5 emerge as the principal signature of the transition from early to late hepatic transcription response to excess dietary fat. The coincidence of the repression of inflammatory, immune and cell proliferation pathways and the induction of metabolic, lipogenic and adipogenic pathways prompted the hypothesis that these events may be interdependent and their swap relevant for driving the transition from a stressed to pathological hepatic state. The relationships between inflammatory and metabolic processes and the key regulators controlling them were further investigated using biological network analysis.

### Network analysis of the HF-responsive genes: interplay between PPARγ and NF-κB regulatory modules

**Figure 4:**
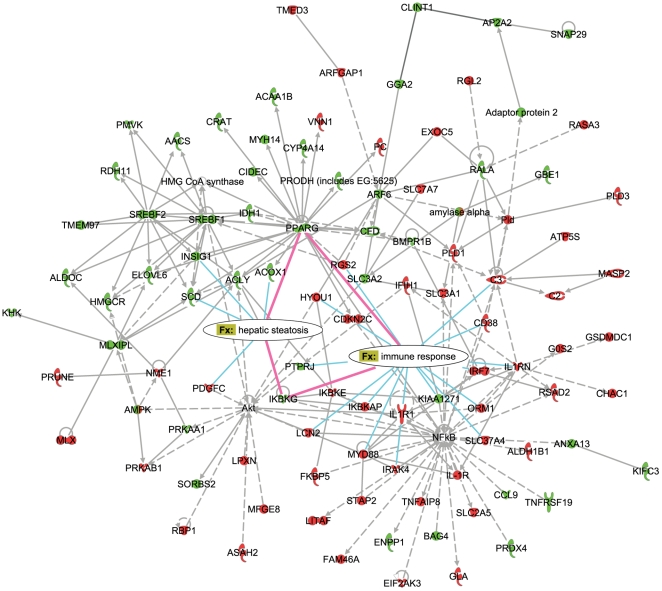
Molecular network underlying hepatic response to high-fat diets (day 3). The global molecular associations of the high-fat responsive genes are functionally characterized and divided into networks based on the functions and/or diseases that are most significant to the network objects (Ingenuity Pathway Analysis). Depicted is the result of merging the network 1 (Immune Response, Tissue Development, Skeletal and Muscular System Development and Function), network 2 (Cellular Development, Connective Tissue Development and Function, Lipid Metabolism) and network 4 (Hepatic System Disease, Liver Steatosis, Cancer). The overrepresented “Function and disease” (Fx) categories “immune response” and “hepatic steatosis” are overlaid onto resulting network, showing which genes (nodes) are directly involved in these processes. The interactions between nodes that are directly connected to both processes are highlighted in pink. Color coding of the nodes corresponds to the direction of gene expression changes at day 3 in HFBT vs. chow diet comparison (upregulated genes are shown in red and downregulated in green).

**Figure 5:**
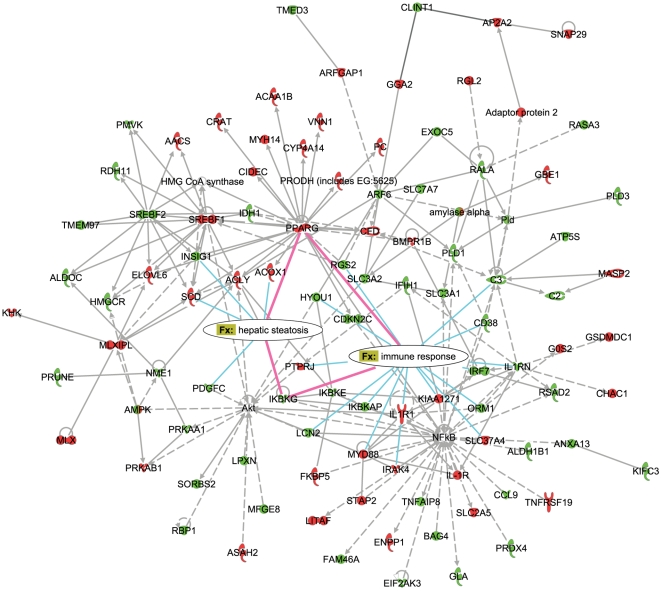
Molecular network underlying hepatic response to high-fat diets (week 12). The equivalent network to that in Figure 4, except that the color coding of the nodes corresponds to the direction of gene expression changes at week 12 in HFBT vs. chow diet comparison.

To further explore the control of and the biological connectivity between the HF-responsive genes, the set of 1663 genes (Figure 1B, 1C, Table S1, Table S2) was used as an input for the network analysis within the Ingenuity Pathway Analysis suite [31]. The networks with the highest significance score (network score equal to or higher than 35) and their associated biological functions are listed in the Table 2. To focus on the interactions between the processes identified as crucial for the transition from early to late hepatic response to excess dietary fat, networks related to immune response, lipid metabolism and hepatic steatosis (networks 1, 2 and 4) were merged for further examination (Figure 4, 5). The network number was limited to three to restrict the size and facilitate the clarity of the resulting network.

**Table 2:** Top scoring networks among the high-fat responsive genes (Ingenuity Pathway Analysis).

Network number	Score*	Focus Genes**	Top Functions
1	40	33	Immune Response, Tissue Development, Skeletal and Muscular System Development and Function
2	40	33	Cellular Development, Connective Tissue Development and Function, Lipid Metabolism
3	37	32	Protein Synthesis, Genetic Disorder, Neurological Disease
4	35	31	Hepatic System Disease, Liver Steatosis, Cancer
5	35	31	Endocrine System Development and Function, Lipid Metabolism, Small Molecule Biochemistry

*Score is the negative exponent of the p-value representing the likelihood that the network eligible molecules that are part of a network are found therein by random chance alone.

**Focus Genes is the number of network eligible genes from the input list represented in the network.

The analysis identified several major regulators of the cellular response to high-fat diets appearing as the hubs in the resulting network: PPARγ, SREBF1 and SREBF2 - regulators of lipid, fatty acid and cholesterol metabolism, NF-κB – regulator of immune response and Akt – regulator of cell growth, proliferation and differentiation. Aside from the constant transcriptional shutdown of the SREBF2 local subnetwork that regulates cholesterol biosynthesis, the majority of network components show a characteristic swap in transcription response between early (day 3) and late (week 12) phase of the time-course (Figure 4, 5).

To further investigate relations between the network components that show the observed swap in transcription response and to characterize their associated functions in more detail, we examined “Function and disease” categories that are overrepresented in the network. By overlaying the most significant categories over the network, the categories “hepatic steatosis” (p-value 1.41E-08) and “immune response” (p-value 5.85E-07) were identified as the best representatives of processes that are reciprocally regulated during the early and the late phase of high-fat response. In addition to the largely reciprocal regulation, these processes are interconnected via few key network components (pink lines, Figure 4, 5). The gene expression of nearly all the network components involved in the promotion of inflammation and immune response is induced during the early phase and repressed during the late phase of the HF response, including NF-κB regulatory factors, interferon, cytokine and chemokine signaling molecules, acute phase response reactants and complement components. In contrast, network members promoting development of hepatic steatosis, such as *PPARγ*, *SCD*, *SREBF1*, *ACOX1*, *CIDEC* and *CFD* (adipsin) are repressed during early phase and induced during the late phase of hepatic response to HF diets. The exception to the observed global repression of inflammatory and immune response in the late phase are interleukin-1 pathway components and, somewhat, the acute phase reactants that show statistically insignificant, low-grade re-induction at week 12 and week 8, respectively. This indicates that a modest fraction of the inflammatory response, likely mediated by JNK/AP-1 pathway parallel to NF-κB signaling escapes the global repression [32], [33].

**Figure 6:**
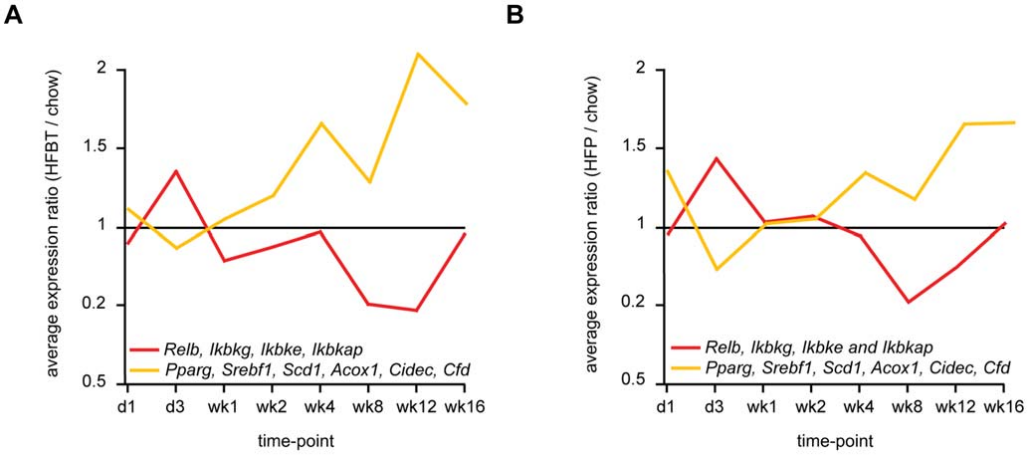
Reciprocal activation of regulators of an inflammatory and steatotic transcriptional programs during high-fat feeding time-course. The average gene expression profiles of NF-kB regulators (*RelB, IKBKG, IKBKE, IKBKAP*) (red line) and PPARγ/hepatic steatosis-associated genes (*PPARγ, SREBF1, SCD1, ACOX1, CIDEC, CFD*) (yellow line) during the 16-week high-fat feeding time-course. (A) HFBT vs. chow diet. (B) HFP vs. chow diet.

Particularly interesting are the network members that are associated with both inflammatory and steatotic transcriptional modules, namely *PPARγ* and *IKBKG* (inhibitor of kappaB kinase gamma). It has been previously shown that PPARγ antagonizes inflammatory responses by a transrepression of NF-κB regulators and that its hepatic activation leads to the development of liver steatosis [11], [12], [34]–[38]. In contrast, IKBKG, also known as NF-κB essential modulator (NEMO), is required for the activation of the NF-κB complex by proinflammatory stimuli, and it has recently been recognized as suppressor of hepatic steatosis, possibly through the negative interaction with PPARs [39]–[42]. The gene expression data show significant repression of *PPARγ* during the early phase of the high-fat response. In contrast, at the mid and/or late phases, *PPARγ* and its target genes are induced, while *NEMO* is simultaneously significantly repressed (Figure 6, Table S2). This reciprocal transcriptional activity proposes an appealing model where the direct trans-repression between PPARγ and NEMO/NF-κB regulators may occur, controlling the transition from early to late hepatic response to HF diets. Evidently, further biochemical studies are required to confirm the suggested temporal transrepression.

### Transition from hepatic inflammation to steatosis is reflected by inflammatory plasma proteins and hepatic triglyceride levels

**Figure 7:**
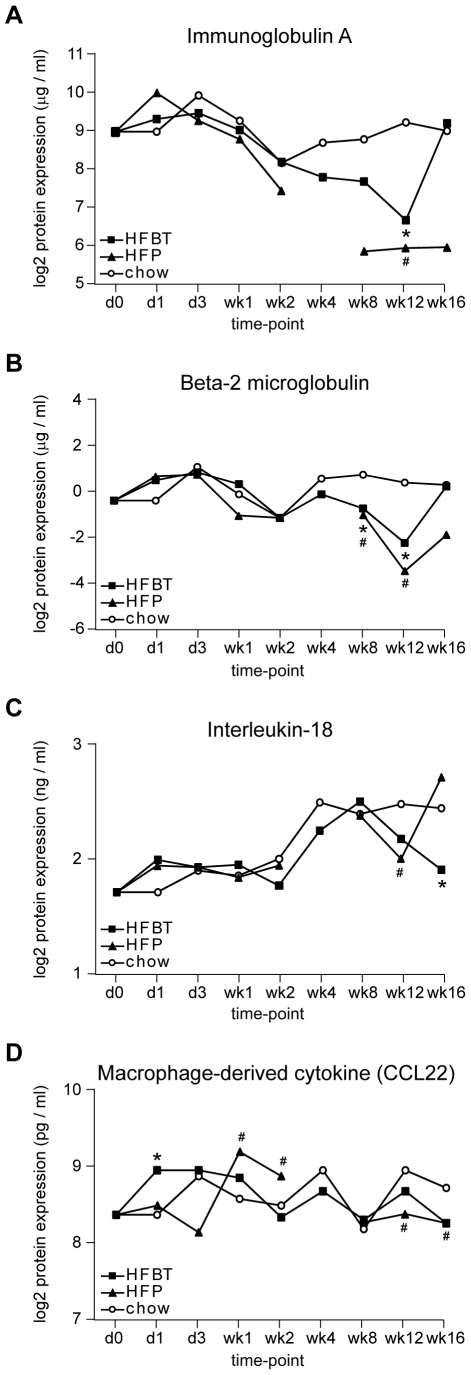
Expression changes of plasma proteins caused by HFBT and HFP high-fat diets. Expression changes of a subset of inflammatory plasma proteins that show trend of early activation coupled with late repression compared to the control condition during the 16-week high-fat (HF) feeding time-course, as measured by multiplex immunoassay. Plotted are average protein expression levels per time point in mice fed chow, HFBT and HFP diets. Statistically significant changes in protein expression of HFBT and HFP fed mice compared to chow fed mice per time-point are marked with asterisk and hash symbol, respectively (p value<0.05). All four proteins are associated with NF-κB activation. (A) Immunoglobulin A, protein that activates NF-κB. (B–D) Beta-2 microglobulin, Interleukin-18 and Macrophage-derived chemokine (CCL22), proteins activated by NF-κB.

The transition from an inflammatory (early) to steatotic (late) transcriptional program in livers of ApoE3L mice observed by gene expression profiling is supported by expression of plasma proteins and liver triglyceride content. A series of inflammatory plasma proteins were quantified by multiplex immunoassay during the complete high-fat feeding time-course (Materials and methods). The transient activation of many inflammation-related proteins has been observed during the early phase of the time-course (data not shown). The levels of plasma proteins likely reflect a systems response of multiple organs to excess dietary fat. Nevertheless, the trend of early activation coupled with late repression, which has been observed in the expression of many hepatic inflammatory genes when HF diets are compared to chow diet, can also be found in the expression of NF-κB-related plasma inflammatory proteins. Examples of such proteins are NF-κB activating protein Immunoglobulin A (Figure 7A) and NF-κB activated proteins Beta-2 Microglobulin, Interleukin-18 and Macrophage-Derived Chemokine (CCL22) (Figure 7B-D) [43]–[47]. The upregulation of Interleukin-18 at week 4 and week 8 under all three dietary conditions is followed by HF-specific repression at the two last time-points (Figure 7C).

**Figure 8:**
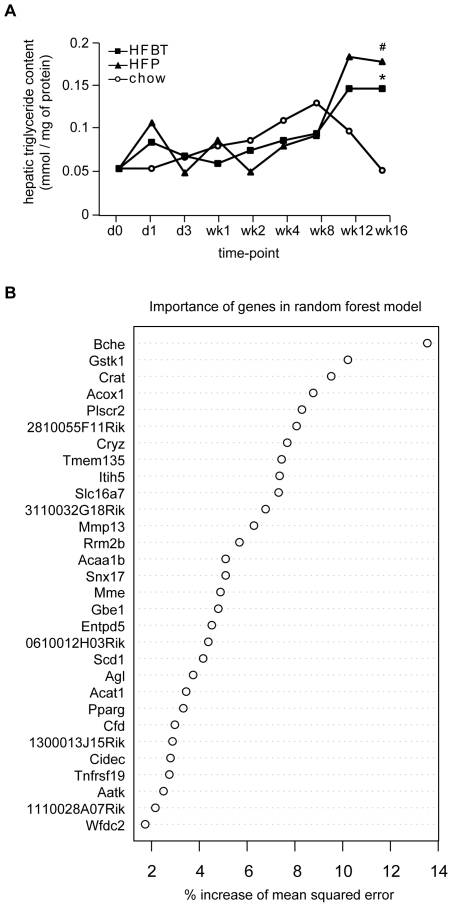
Regression analysis of gene expression and hepatic triglyceride content in chow and high-fat-fed ApoE3L mice. (A) Changes in hepatic triglyceride (TG) content induced by HFBT and HFP high-fat diets and a control (chow) diet during the 16-week time course. Plotted are average values of hepatic triglyceride levels per time point in each of the three diets. Statistically significant increase (p≤0.01) in hepatic TG content of HFBT and HFP fed mice compared to chow fed mice (marked with asterisk and hash symbol, respectively) was observed at the time-point 16 weeks, indicating development of hepatic steatosis. (B) The most important genes for the prediction of hepatic triglyceride levels, as assessed by Random Forests Regression analysis. The expression of 1663 high-fat responsive genes and the hepatic triglyceride levels in each animal were used as an input for the regression analysis. The plot shows the importance of the top 30 genes in the Random Forest regression model. The importance of a gene in the model is expressed as the increase of Mean Squared Error (MSE) when the gene is excluded from the analysis. Higher the percentage of increase of MSE, the more important the particular gene is for the prediction of the hepatic TG levels. The genes identified as steatosis-associated by the network analysis, such as *ACOX1*, *SCD*, *PPARγ*, *CFD* and *CIDEC* are also discovered among the top 30 genes resulting from the regression analysis of gene expression and hepatic TG levels.

The activation of the steatotic transcriptional program during the late phase of the high-fat feeding time-course observed by transcriptome analysis harmonizes with the increased hepatic triglyceride content at the late time-points (Figure 8A). To identify genes whose expression is most predictive of hepatic fat accumulation under high-fat dietary conditions, liver triglyceride content and expression of 1663 high-fat responsive genes in each animal were used to perform regression analysis by random forest modeling [48], [49]. The genes with highest importance in the resulting model (n = 30) are shown in Figure 8B. The genes we identified as steatosis-associated by the network analysis (Figure 4, Figure 5), such as *ACOX1*, *SCD*, *PPARγ*, *CFD* and *CIDEC* were re-discovered by the regression analysis. Also, matrix metallopeptidase 13 (*MMP13*) implicated in liver fibrosis and *ENTPD5*, associated with hepatopathy and hepatocellular tumors were identified by the regression approach 50, 51. In addition to the known markers of hepatic pathology, random forest modeling uncovered putative novel candidate genes whose expression may predict the development of hepatic steatosis. This includes fatty acid metabolism associated genes (*CRAT, ACAA1b* and *ACAT*), matrix endopeptidase related proteins (*ITIH5*, *MME*) and several genes of unknown function. The gene identified as the best predictor of hepatic steatosis by the regression analysis, butyrylcholinesterase (*BCHE*) has been mostly studied in the context of its effect on the brain's cholinergic system [52]. However, there is evidence showing that serum butyrylcholinesterase is associated with adiposity, serum lipid profile and insulin resistance in humans [53]. Therefore, *BCHE* may be the potential marker of interest for further investigation of the hepatosteatosis development.

Together, inflammatory plasma proteome and hepatic triglyceride content support findings obtained by mRNA expression analysis, further substantiating the proposed shift between the hepatic inflammatory and steatotic transcriptional program during prolonged exposure to high-fat diets.

## Discussion

### Hepatic pathophysiological changes induced by short- and long-term exposure to excess dietary fat

In this study, we focused on investigating the molecular mechanisms underlying the onset and progression of the metabolic syndrome during a 16-week high-fat feeding time-course in ApoE3L transgenic mice. The changes in hepatic transcriptome revealed that the adaptation to excess dietary fat proceeds in three phases: early, mid and late. The early (day 1 to week 1) and the late (week 8 to week 16) phases are characterized by the most prominent, and often reciprocal peaks in gene expression changes.

During the early phase, the initial sensing of the fat overload triggers the cellular stress response, characterized by activation of acute phase reactants, inflammatory and immune response (Figure 2, 3, 4). The deregulation of the cell cycle and certain apoptotic genes during the early phase of high fat feeding resemble hepatic regeneration response, further suggesting compromised liver integrity. Additionally, the immediate perturbation occurs in mitochondrial, microsomal and peroxisomal oxido-reductive processes, including the severe repression of specific members of cytochrome P450 family and glutathione transferase genes (Figure 2, 3, Table S2). The imbalance in oxido-reductive processes accompanied with a diminished protective function of glutathione transferases may create conditions of elevated liver sensitivity to oxidative damage [54]. The hypersensitivity to oxidative stress could synergize with the lipotoxic stress, manifested by the activation of unfolded protein response (UPR), endoplasmatic reticulum (ER) stress and DNA damage response (Figure 2, 3). Apart from the stress response, the early phase of the hepatic transcriptional response to high-fat diets is also characterized by metabolic adaptation. Of all the pathways involved in lipid metabolism, early activation is exclusively observed in lipid/lipoprotein binding and transport (Figure 2, 3). This suggests that the primary, short-term metabolic adjustment may involve hepatic elimination of fatty acid surplus by means of its excretion in the form of very low-density lipoprotein (VLDL) particles. Cholesterol efflux and biliary secretion are also immediately activated, coupled with repression of cholesterol biosynthesis (Figure 2, 3).

The major signature of the late phase of the hepatic adaptation to excess dietary fat is the transcriptional induction of nearly all aspects of lipid metabolism, including those regulated by PPARα (lipolysis, fatty acid beta-oxidation), PPARγ (adipogenesis and adipogenic transformation, lipogenesis, lipid accumulation, lipid uptake) and SREBP1 (lipogenesis, fatty acid synthesis, fatty acid desaturation, fatty acid elongation) (Figure 2, 3, 5). The need for long-term lipid management is, thus, likely resolved by an activation of both lipolysis and hepatic lipid storage. The pro-steatotic transcriptional program, manifested in the activation of lipogenic, adipogenic and lipid accumulation pathways and the activation of genes such as *PPARγ* gene itself, stearoyl-CoA desaturase 1 (*SCD1*) and *CIDEC* (*Fsp27*) (Figure 2, 3, 5, 6) suggest that the adipogenic transformation of hepatocytes and the development of liver steatosis may occur during the late phase of HF feeding [34]–[37], [55]. This finding is supported by the increase of total hepatic triglycerides in the late phase of the time-course and their significantly elevated levels at the week 16 in mice fed HF diets compared to chow fed mice (Figure 8A). The incidence of hepatic steatosis under the similar experimental conditions has also been reported in the literature [21], [35]. The regression analysis of association between hepatic gene expression and the lipid accumulation identified known and novel genes that may be valuable as predictors of hepatosteatosis development.

The observed hepatic adaptation to excess dietary fat occurs similarly in investigated beef tallow- (HFBT) and palm oil- fat (HFP) based diets. Nevertheless, specific differences in the gene expression response to these two diets do exist. This is particularly evident in the expression of pathways related to energy metabolism during the early and the long-term adaptation to high-fat feeding. In general, mice fed HFP diet show more pronounced transient induction of these pathways at the very beginning of the time course, but fail to activate them as efficiently as the mice fed HFBT diet at the late phase of the time-course (Figure 3). These differences in processes that are relevant for energy expenditure may account for a higher increase in body weight (Figure 1A) and significantly higher whole-body insulin resistance (data not shown) of HFP-fed mice compared to HFBT-fed mice at the end of the time-course. This observation may be relevant in the context of dietary recommendations, suggesting that the excess of palm oil-fat based diet may be at least as harmful, if not more so, than the cholesterol-containing beef tallow fat-based diet.

### The hypothesized mechanism controlling the switch from an inflammatory to steatotic hepatic state during the high-fat feeding response

The majority of pathways affected by the high-fat diets exhibit opposite regulation during the early and the late phase of the high-fat feeding time-course. This synchronous swap of the major functional signatures between the early and the late phase and the fact that the key controllers of the reciprocally regulated processes (as revealed by the network analysis (Figure 4, Figure 5)) are implicated in the mutual repression suggest that the regulatory exchange may occur via tightly controlled reactions, limited to few master regulators.

The NF-κB and Akt regulators, the key controllers of the pathways showing early activation/late repression expression mode (i.e. inflammation, immune response, cell proliferation and cell differentiation), have been previously reported to act synergistically [56]–[59]. Similarly, PPARγ and SREBP1, identified in our study as the key regulators of pathways in the early repression/late activation transcriptional module are co-acting in regulating lipid metabolism and adipogenesis [60], [61]. In contrast to the synergistic activities within these transcriptional modules, there is emerging evidence of the antagonistic activity between them, particularly regarding NF-κB and PPAR regulators. There is limited evidence for the NF-κB mediated repression of PPARγ [62]. In turn, multiple mechanisms by which PPARs inhibit inflammatory gene expression through interference with NF-κB signaling have been reported (reviewed in [11], [15], [63]). Several studies investigated the inhibitory effect of PPARγ and its agonists on the activity of the inhibitor of nuclear factor κB kinase (IKK) complex [64]–[66]. The IKBKG (NF-κB essential modulator, NEMO) is the subunit of the IKK complex indispensable for the activation of NF-κB [42]. Considering its critical role, we propose the model in which down-regulation of *NEMO* in the mid and the late phases of HF response could be the most important regulatory event controlling the shut-down of NF-κB driven inflammatory response at the switch point between stressed and pathological hepatic state. In addition to *NEMO*, other two IKK related genes (*IKBKE, IKBKAP*) and the gene coding for NF-κB subunit RelB also show characteristic early induction and late repression transcription mode (Figure 6, Table S2). The coincident opposite transcriptional activity of *PPARγ* and its target pathways suggests that the two regulatory events may be interdependent (Figure 6). This hypothesis is supported by the recent studies showing that NEMO has an essential physiological role in preventing the spontaneous development of hepatic steatosis preceding hepatocellular carcinoma and that the hepatic ablation of NEMO in mice fed HF diet increases *PPARγ* mRNA levels and aggravates hepatic steatosis [39], [40]. The suggested mechanism of the mutual repression of PPAR and NEMO/NF-κB regulators during their coordination of the transition from early to the late phase of HF-response remains to be confirmed by biochemical studies.

### The relevance of the proposed model of the transition from high-fat induced metabolic stress to metabolic syndrome in ApoE3L mice

**Figure 9:**
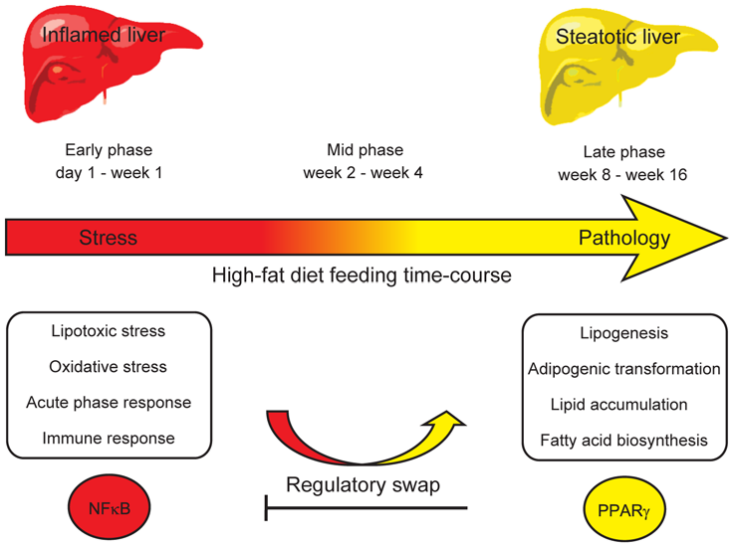
Model of the hepatic physiological response to high-fat diets during the 16-week time-course. The summary of a proposed model for the hepatic physiological changes in response to high-fat diets during the 16-week time-course in ApoE3L mice. The initial perturbation of hepatic homeostasis by excess dietary fat triggers the stress response largely controlled by NF-κB and Akt regulators and manifested in activation of acute phase response, inflammation, immune response, hepatic regeneration-like response and lipotoxicity (day 1 to week 1). Upon prolonged high-fat feeding, liver fails to regain the basal state and consequently shifts to pathological state controlled by PPAR and SREBP regulators and characterized by hepatic lipid accumulation and adipogenic transformation, indicative of hepatic steatosis (late phase, week 8 to week 16). The flagship processes induced at the early and at the late phase are shown in boxes. The transition between the stressed and the pathological hepatic state may be controlled by trans-inhibitory interactions between NF-κB and PPARγ regulators, resulting in the tradeoff between inflammatory and steatotic transcription programs (mid phase, week 2 to week 4). On the systems level, the activation of steatotic program is followed by other metabolic syndrome associated pathologies such as obesity and whole-body insulin resistance.

The dynamic functional landscape of the hepatic transcriptional adaptation to excess dietary fat during the 16-week time-course suggests a model in which sequential physiological changes underlie the transition from metabolic stress to metabolic syndrome, summarized in Figure 9. These results provide novel insight into the delicate cross-talk between inflammation and lipid metabolism in controlling the progression of metabolic disease development. It is important to note that the late repression of the hepatic NF-κB driven inflammatory/immune response may not seem in line with the established model of the obesity-associated inflammation, well studied in the adipose tissue [10], [67]. The previously shown association of inflammatory signaling pathways with obesity and hepatic steatosis is the most prominent feature observed in our data. Nevertheless, the assumption that, similar to the situation in adipose tissue, hepatic inflammation is secondary to hepatic steatosis is not supported by our findings [68], [69]. In fact, our results show that the temporal order of events contradicts this assumption, at least during the examined time frame and in our mouse model. Extending the duration of treatment and/or increasing amount of excess dietary fat would likely provoke transitions to further grades of severity in hepatic pathology such as hepatosteatitis, fibrosis, cirrhosis and hepatocellular carcinoma. To fully understand the complex relationship between inflammation and metabolic syndrome, information originating from different organs and at various time points needs to be considered on the systems level [69].

Finally, the identified central role of ppar and nemo/nf-κb regulators in coordinating the onset and progression of metabolic syndrome may have important implications in treatment of the disease. currently, pparγ ligands are used in clinics for their anti-inflammatory and insulin-sensitizing effects in diseases such as psoriasis, atherosclerosis, inflammatory bowel disease and type 2 diabetes [13], [14], [70]–[74]. our results suggest that pparγ activation could also have negative effects in disorders where the cross-talk between inflammation and lipid metabolism is essential to the development of pathogenesis, as it is in diet-induced metabolic syndrome. the activation of pparγ may inhibit nf-κb and therefore suppress inflammation, but in turn evoke transition to pathological state, in this case hepatic steatosis. despite the apparent harmful effects of inflammation, such as triggering insulin resistance, its protective physiological role in preventing transitions to even less preferable system states should not be neglected. thus, the tradeoff between the beneficial and harmful effects of altered pparγ activity should be carefully considered when using pparγ ligands and nf-κb-inhibiting agents to ameliorate metabolic syndrome associated pathologies. the presented findings demonstrate the use of high-throughput dataset analyses as a starting point for generating testable hypotheses that may open new avenues for dietary prevention strategies, clinical research and pharmaceutical therapies.

## Materials and Methods

### Ethics Statement

Animal experiments were approved by the Institutional Animal Care and Use Committee of the Netherlands Organization for Applied Scientific Research (TNO) and were in compliance with European Community specifications regarding the use of laboratory animals.

### Animals and diets

The study involved 186 male ApolipoproteinE3-Leiden transgenic mice [24] at 14±2 weeks of age. Apolipoprotein E3-Leiden (ApoE3L) transgenic mice display lipoprotein profile that closely resembles that of humans and develop human-like dysbetalipoproteinemia and atherosclerotic lesions when fed Western-type diets. The age of 14 weeks was chosen as optimal because the animals are considered adult at that stage (neither juvenile at early time points nor aged at the late time points of the study). The first (control) group of ApoE3L mice was fed standard chow diet (RM3 (E) DU; Special Diet Services, Witham, Essex, UK), the second group of mice was fed a high-fat diet based on animal fats (HFBT, beef tallow fat high-fat diet +0.25% cholesterol, also referred to as Western-type diet, Arie Blok BV, The Netherlands) and the third group of mice was fed a high-fat diet based on plant fats (HFP, palm oil high-fat diet based on open source D12451 SDS diet in which lard is replaced with palm oil, Research Diet Services, Woerden, The Netherlands). The macronutrient content and the fatty acid composition of chow and high-fat diets are provided in Supporting .

### Experimental design and sample preparation

From three weeks prior to diet intervention onwards, all animals were fed a standard chow diet. At the beginning of the study, mice were divided into three groups: (1) control group fed chow diet, (2) group fed HFBT diet and (3) group fed HFP diet. Because the interest of the study was to asses effects of high-fat diets under physiological conditions, animals were fed *ad libitum*. Series of control experiments employing a metabolic cage setup showed that C57Bl/6 mice, the genetic background of the APOE3L mice, have isocaloric food intake when fed low fat and HFBT and HFP diets. The light cycles were identical for all animals. For mRNA expression profiling, six mice from each diet group (total n = 150) were sacrificed at time points 0 days (chow only), 1 day, 3 days, 1, 2, 4, 8, 12 and 16 weeks, their livers were dissected after 4 hour fasting period (typically from 9 h AM to 1 h PM), snap frozen in liquid nitrogen and stored at −80°C until further processing.

### RNA isolation, labeling and hybridization to microarrays

Total RNA was isolated using TRIzol reagent (Invitrogen, Breda, The Netherlands) according to the manufacturer's instructions. RNA was treated with DNAse and purified using the SV total RNA isolation system (Promega, Leiden, The Netherlands). Concentrations and purity of RNA samples were determined on a NanoDrop ND-1000 spectrophotometer (Isogen, Maarssen, The Netherlands). RNA integrity was checked on an Agilent 2100 bioanalyzer (Agilent Technologies, Amsterdam, The Netherlands) with 6000 Nano Chips according to the manufacturer's instructions. RNA was considered as suitable for array hybridization only if samples exhibited intact bands corresponding to the 18S and 28S ribosomal RNA subunits, displayed no chromosomal peaks or RNA degradation products, and had a RNA integrity number (RIN) >8. Applying this criterion, 142 RNA samples were used for hybridization to microarrays, including 5 to 6 biological replicate samples per diet, per time-point. RNA samples were hybridized to NuGO Affymetrix Mouse GeneChip arrays (NuGO_Mm1a520177) containing 23865 probesets including 73 control probesets [26]. Arrays were scanned on a GeneChip Scanner 3000 7G (Affymetrix). Detailed methods for labeling, hybridizations to the arrays and scanning are described in the eukaryotic section of the GeneChip Expression Analysis Technical Manual, Revision 3, from Affymetrix, and are available upon request. The gene expression data are made available via ArrayExpress repository [75].

### Data preprocessing, differential expression analysis and Gene Set Enrichment Analysis

Quality control of microarray data, normalization, differential expression analysis and Gene Set Enrichment Analysis [30] were performed using packages from the R/Bioconductor project [76], [77] through the Management and Analysis Database for MicroArray eXperiments (MADMAX) analysis pipeline [78].

Quality control of the hybridized microarrays was performed using simpleaffy and affyplm packages. Upon rigorous examination of the resulting diagnostic plots, 116 microarrays of the supreme quality were taken for the further analysis. This resulted in analysis of 3 to 6 biological replicate samples per diet, per time-point. Gene expression estimates were calculated using the library GC-RMA, employing the empirical Bayes approach for background correction followed by quantile normalization. The custom MBNI CDF-file (MmNuGOMm1a520177 version 9.0.1), available at http://brainarray.mbni.med.umich.edu/Brainarray/Database/CustomCDF/CDF_download_v9.asp and http://nugo-r.bioinformatics.nl/NuGO_R.html [79], [80] was used to re-annotate the probes to new probesets, remove poor quality probes and derive unique signal values for different probesets representing the same gene. This resulted in gene expression values for 15105 genes with unique identifiers.

Differentially expressed genes between control and each of treatment groups per time point, as well as between each of time points and day 0, were identified using the limma package, applying linear models and moderated t-statistics that implement empirical Bayes regularization of standard errors [27]. False discovery rate of 10% (q-value<0.1) was used as a threshold for significance of differential expression. Significance of the overlap between differentially expressed genes in HFBT and HFP conditions (Figure 1B) was calculated by hypergeometric distribution.

The t-test values of differential expression between control and each of treatment groups per time point calculated using limma package were used as the input for the PreRanked scoring method within the Gene Set Enrichment Analysis (GSEA). Gene sets collection included 880 gene sets compiled from MSigDB C2, Biocarta, Kyoto Encyclopedia of Genes and Genomes (KEGG) and GenMAPP databases as well as the expert curated gene sets. Detailed information about gene sets used for GSEA analysis, including source websites is available upon request. Gene set size filter (min = 15, max = 500) resulted in filtering out 405 of 880 gene sets. The number of permutations for was set to 1000. Gene sets are considered significantly enriched at false discovery rate (FDR) smaller than 10% (q-value<0.1). In total, 314 gene sets are identified as significantly enriched in at least 1 of 16 comparisons (HFBT vs. chow and HFP vs. chow per time-point). Normalized enrichment scores (NES) of significantly enriched pathways and the corresponding FDR q-values across all experimental conditions are available upon request.

### Hierarchical clustering and data visualization

Hierarchical clustering and visualization of gene expression changes in Figures 1C, 2 and 6 were performed in GeneSpring GX 7.3.1 software (Agilent), using average gene expression values under HFBT and HFP conditions divided by the median of chow gene expression, per time-point. Hierarchical clustering and visualization of NES values in Figure 3 were performed using HierarchicalClusteringViewer and HeatmapViewer modules within the GenePattern analysis suite [81]–[83].

### Analysis of gene expression profiles of HF-responsive genes

Temporal analysis of the gene expression profiles where each of HF groups is compared to chow per time point (Figure S2, S3, S4 and S5) was performed using Smoothing Spline Clustering algorithm [28] through the R computing environment [76], applying the settings nchain = 5 and nclust = 24. Setting the number of clusters to 24 yielded Bayesian information criterion (BIC) values that were at the bottom of the U-shaped BIC curve (i.e. closely approaching the optimum), while resulting in images of reasonable complexity. As input values, average log_2_ ratios of 1663 high-fat responsive genes were used (HFBT and HFP divided by median of chow per time point). To facilitate visualization of temporal trends, the starting time point (t = 0, only chow fed mice) was also included in the analysis.

### Overrepresentation analysis of functional categories

Identification of overrepresented functional categories among 1663 high-fat responsive genes and their grouping into functionally related clusters was performed using DAVID Functional Annotation Clustering tool [29]. The analysis was performed using Gene Ontology, Protein domains, Pathways and Functional categories according to the default settings (Table S3, version date July 2007). Representative functional categories from the most statistically significant clusters are manually selected and listed in Figure 2. Equivalent analysis is performed using separately upregulated (922 genes) and downregulated (786 genes) high-fat responsive genes (Table S3, version date June 2009).

### Network analysis

The network analysis was generated through the use of Ingenuity Pathways Analysis (version date March 2008) [31]. The data set containing gene identifiers and corresponding expression values for 1663 high-fat responsive genes was uploaded into the application. Of 1663 gene identifiers, 1660 were successfully mapped to its corresponding gene objects in the Ingenuity Pathways Knowledge Base and 1303 were identified as network eligible. These genes were overlaid onto a global molecular network developed from information contained in the Ingenuity Pathways Knowledge Base. Networks were then algorithmically generated and graphically represented based on connectivity of genes. The Functional Analysis of the network identified the biological functions and/or diseases that were most significant to the genes in the network (version date June 2009). Fischer's exact test was used to calculate a p-value determining the probability that each biological function and/or disease assigned to that network is due to chance alone. Genes or gene products are represented as nodes, and the biological relationship between two nodes is represented as an edge (line). The node color indicates up- (red) or down- (green) regulation. The direction of average expression changes in HFBT vs. chow comparisons (Table S2) at day 3 and week 12 was used for color coding in Figure 4 and Figure 5, respectively.

### Accession numbers

For the microarray experiments described in this study, MIAME compliant protocols and datasets in Tab2MAGE are accessible from ArrayExpress microarray data repository [75] with the accession number E-TABM-553.

### Plasma proteins and hepatic triglyceride content analysis

Plasma proteins were quantified by multiplex immunoassay measurements at Rules Based Medicine (Rules Based Medicine, Inc., Austin, Texas, USA). Plasma antigens immunoassay panel included in the Rodent Multi-Analyte Profile was used for measurement of expression levels of 58 proteins (RodentMAP version 2 antigen panel). Of these, 47 proteins had sufficient detectability of the expression signals and were included in the further analysis. Statistical significance of protein expression in HFBT and HFP fed mice compared to chow fed mice per time-point (marked with asterisk and hash symbol, respectively) was assessed by t-test. The p value of 0.05 was used as a threshold for significance.

Liver lipid content was defined as total triglyceride content (mmol) per mg of protein. Extraction was performed using a modified Bligh and Dyer extraction protocol, optimized for steatotic liver material. Triglyceride content was measured enzymatically using the Roche TG kit (Roche, cat. No. 11488872216). Protein content was measured by BCA analysis (BCA protein Assay Kit, Pierce, cat. No. 23225). Statistical significance of hepatic triglyceride content in HFBT and HFP fed mice compared to chow fed mice per time-point (marked with asterisk and hash symbol, respectively) was assessed by t-test. The p value of 0.01 was used as a threshold for significance.

### Regression analysis of gene expression and hepatic triglyceride content

To determine the relation between gene expression data and triglyceride levels in the liver tissue, Random Forests regression was used through randomForest package of R statistical computing environment [48], [49], [76], [84]. The gene expression values of 1663 high-fat responsive genes and the hepatic triglyceride levels of the matching animals at each time-point were used as an input for the analysis. In Random Forests regression, a group of regression trees is used for the model. In every individual regression tree, the data are split at each node until all objects are in a single leaf. For each tree only a subset of the data is used and at each node only a subset of the variables is used to make the split. By intentionally incorporating only part of the data for each tree, the data which are not used to generate the tree can be used to assess its performance. The term used for this measure is the Out Of Box (OOB) error. Besides the OOB measure for the overall model, it is possible to asses the importance of individual variables in the model. Here we used the increase of Mean Squared Error (MSE). The Mean Squared Error is a general measure for the overall fit of a model and its increase measures the importance of individual variables (in this case genes) when they are excluded from the analyses. The higher the increase of the MSE when a gene is excluded from the analysis, the more important that particular gene is for the fit of the model (and for the prediction of triglyceride levels in liver). The MSE of the Random Forests regression model presented here is 49.9 percent. The top 30 genes that have the highest increase of MSE and therefore provide best prediction of triglyceride levels are shown in Figure 8B. The term prediction is used here to refer to the ability to predict the triglyceride levels as they were measured on the basis of gene expression, rather than in the sense of prognostic for the future progress of steatosis in time.

Random Forests is an improved Classification and Regression Trees (CART) method. It grows many classification trees or regression trees, hence the name ‘Forests’. For quantitative outcomes the forest is made of regression trees, where the tree predictor is the mean value of the training set observations in each terminal leaf. In our application of random forests the outcomes are quantitative, therefore the regression algorithm and not classification was used. Every tree is built using a deterministic algorithm and the trees are different owing to two factors. First, at each node, a best split is chosen from a random subset of the predictors rather than all of them. Second, every tree is built using a bootstrap sample of the observations. The out-of-bag (OOB) data, one-third of the observations, are then used to estimate the prediction accuracy. Unlike other tree algorithms, no pruning, trimming of the fully grown tree, is involved. Each observation is assigned to a leaf, the terminal node of a tree, according to the order and values of the predictor variables. For a particular tree, the predictions for observations are given only for the OOB data. The Random Forest predictor is computed by averaging the tree predictors over trees for which the given observation was OOB. Because the prediction for an observation is based on trees grown without the observation, an idea akin to cross-validation, the estimated errors are unbiased and the data were not divided in test and training sets.

## Supporting Information

Figure S1 Temporal changes in gene expression for HFBT, HFP and chow diet over the 16-week period compared to day 0. The number of statistically significant differentially expressed genes (DEG) identified by pairwise comparison of each time-point versus day 0 (limma, FDR<0.1) in chow, HFBT and HFP dietary conditions. Venn diagram shows overlap of total (in any of time points) number of DEGs in the three diets.
(0.37 MB PDF)

Figure S2 Temporal gene expression profiles (HFBT, raw curves). The results of Smoothing Spline Clustering analysis [25] for 1663 high-fat responsive genes. The genes are grouped into 24 clusters according to their temporal expression profiles. The vertical axis represents the expression ratios and the horizontal axis the time points 1 to 9 (day 0, day 1, day 3, week 1, week 2, week 4, week 8, week 12 and week 16). Figure S2 corresponds to the HFBT experimental conditions and raw expression ratio values.
(0.07 MB PDF)

Figure S3 Temporal gene expression profiles (HFBT, mean curves). The results of Smoothing Spline Clustering analysis [25] for 1663 high-fat responsive genes. The genes are grouped into 24 clusters according to their temporal expression profiles. The vertical axis represents the expression ratios and the horizontal axis the time points 1 to 9 (day 0, day 1, day 3, week 1, week 2, week 4, week 8, week 12 and week 16). Figure S3 corresponds to the HFBT experimental conditions and mean expression ratio values (and their confidence intervals, in red) of the genes in each cluster.
(0.02 MB PDF)

Figure S4 Temporal gene expression profiles (HFP, raw curves). The results of Smoothing Spline Clustering analysis [25] for 1663 high-fat responsive genes. The genes are grouped into 24 clusters according to their temporal expression profiles. The vertical axis represents the expression ratios and the horizontal axis the time points 1 to 9 (day 0, day 1, day 3, week 1, week 2, week 4, week 8, week 12 and week 16). Figure S4 corresponds to the HFP experimental conditions and raw expression ratio values.
(0.07 MB PDF)

Figure S5 Temporal gene expression profiles (HFP, mean curves). The results of Smoothing Spline Clustering analysis [25] for 1663 high-fat responsive genes. The genes are grouped into 24 clusters according to their temporal expression profiles. The vertical axis represents the expression ratios and the horizontal axis the time points 1 to 9 (day 0, day 1, day 3, week 1, week 2, week 4, week 8, week 12 and week 16). Figure S5 corresponds to the HFP experimental conditions and mean expression ratio values (and their confidence intervals, in red) of the genes in each cluster.
(0.02 MB PDF)

Table S1 Detailed overview of numbers of differentially expressed genes in HFBT vs. chow and HFP vs. chow comparisons (limma, FDR<0.1). The information summarized in Table 1 is extended to a detail to include: (1) number of upregulated genes per diet and time point, (2) number of downregulated genes per diet and time point, (3) total (both up- and downregulated) number of differentially expressed genes per diet and time point, (4) number of overlapping genes between HFBT and HFP diets for each of categories (1)–(3), (5) number of genes in union of HFBT and HFP diets for each of categories (1)–(3) and (6) total (in all time-points) number of differentially expressed genes for categories (1)–(5).
(0.05 MB DOC)

Table S2 Annotation and statistics for 1663 high-fat responsive genes. The list of 1663 genes differentially expressed under the high-fat conditions (false discovery rate q-value<0.1 in either of HFBT versus chow and HFP versus chow comparisons per time-point), including detailed annotation of these genes, expression ratios and the statistics (as determined by limma package) in each of the experimental conditions.
(3.10 MB XLS)

Table S3 Overrepresentation analysis of functional categories among all, upregulated and downregulated HF-responsive genes. The complete results of functional categories overrepresentation analyses and their grouping into functionally related clusters generated using DAVID Functional Annotation Clustering tool [29]. The analyses were performed using (1) all (n = 1663), (2) upregulated (n = 922) and (3) downregulated (n = 786) high-fat responsive genes.
(1.89 MB XLS)

Table S4 The macronutrient content and the fatty acid composition of chow and high-fat diets. The macronutrient content and the fatty acid composition of chow, HFBT and HFP diets.
(0.04 MB DOC)

## References

[1] Kopelman PG (2000). Obesity as a medical problem.. Nature.

[2] Kopelman PG, Grace C (2004). New thoughts on managing obesity.. Gut.

[3] Ogden CL, Yanovski SZ, Carroll MD, Flegal KM (2007). The epidemiology of obesity.. Gastroenterology.

[4] Lissner L, Heitmann BL (1995). Dietary fat and obesity: evidence from epidemiology.. Eur J Clin Nutr.

[5] Buettner R, Scholmerich J, Bollheimer LC (2007). High-fat diets: modeling the metabolic disorders of human obesity in rodents.. Obesity (Silver Spring).

[6] Olefsky J, Reaven GM, Farquhar JW (1974). Effects of weight reduction on obesity. Studies of lipid and carbohydrate metabolism in normal and hyperlipoproteinemic subjects.. J Clin Invest.

[7] Lin S, Thomas TC, Storlien LH, Huang XF (2000). Development of high fat diet-induced obesity and leptin resistance in C57Bl/6J mice.. Int J Obes Relat Metab Disord.

[8] Muller M, Kersten S (2003). Nutrigenomics: goals and strategies.. Nat Rev Genet.

[9] Medzhitov R (2008). Origin and physiological roles of inflammation.. Nature.

[10] Hotamisligil GS (2006). Inflammation and metabolic disorders.. Nature.

[11] Bensinger SJ, Tontonoz P (2008). Integration of metabolism and inflammation by lipid-activated nuclear receptors.. Nature.

[12] Delerive P, De BK, Besnard S, Vanden BW, Peters JM (1999). Peroxisome proliferator-activated receptor alpha negatively regulates the vascular inflammatory gene response by negative cross-talk with transcription factors NF-kappaB and AP-1.. J Biol Chem.

[13] Gervois P, Fruchart JC, Staels B (2007). Drug Insight: mechanisms of action and therapeutic applications for agonists of peroxisome proliferator-activated receptors.. Nat Clin Pract Endocrinol Metab.

[14] Lehmann JM, Moore LB, Smith-Oliver TA, Wilkison WO, Willson TM (1995). An antidiabetic thiazolidinedione is a high affinity ligand for peroxisome proliferator-activated receptor gamma (PPAR gamma).. J Biol Chem.

[15] Straus DS, Glass CK (2007). Anti-inflammatory actions of PPAR ligands: new insights on cellular and molecular mechanisms.. Trends Immunol.

[16] Duivenvoorden I, Teusink B, Rensen PC, Kuipers F, Romijn JA (2005). Acute inhibition of hepatic beta-oxidation in APOE*3Leiden mice does not affect hepatic VLDL secretion or insulin sensitivity.. J Lipid Res.

[17] Gijbels MJ, van der CM, van der Laan LJ, Emeis JJ, Havekes LM (1999). Progression and regression of atherosclerosis in APOE3-Leiden transgenic mice: an immunohistochemical study.. Atherosclerosis.

[18] Hollestelle SC, De Vries MR, Van Keulen JK, Schoneveld AH, Vink A (2004). Toll-like receptor 4 is involved in outward arterial remodeling.. Circulation.

[19] Kleemann R, Verschuren L, van Erk MJ, Nikolsky Y, Cnubben NH (2007). Atherosclerosis and liver inflammation induced by increased dietary cholesterol intake: a combined transcriptomics and metabolomics analysis.. Genome Biol.

[20] Kreeft AJ, Moen CJ, Hofker MH, Frants RR, Vreugdenhil E (2001). Identification of differentially regulated genes in mildly hyperlipidemic ApoE3-Leiden mice by use of serial analysis of gene expression.. Arterioscler Thromb Vasc Biol.

[21] Kreeft AJ, Moen CJ, Porter G, Kasanmoentalib S, Sverdlov R (2005). Genomic analysis of the response of mouse models to high-fat feeding shows a major role of nuclear receptors in the simultaneous regulation of lipid and inflammatory genes.. Atherosclerosis.

[22] van der Hoorn JW, Jukema JW, Bekkers ME, Princen HM, Corda S (2008). Negative effects of rofecoxib treatment on cardiac function after ischemia-reperfusion injury in APOE3Leiden mice are prevented by combined treatment with thromboxane prostanoid-receptor antagonist S18886 (terutroban).. Crit Care Med.

[23] Wouters K, Shiri-Sverdlov R, van Gorp PJ, van BM, Hofker MH (2005). Understanding hyperlipidemia and atherosclerosis: lessons from genetically modified apoe and ldlr mice.. Clin Chem Lab Med.

[24] van den Maagdenberg AM, Hofker MH, Krimpenfort PJ, de BI, van VB (1993). Transgenic mice carrying the apolipoprotein E3-Leiden gene exhibit hyperlipoproteinemia.. J Biol Chem.

[25] van Vlijmen BJ, van den Maagdenberg AM, Gijbels MJ, van der BH, HogenEsch H (1994). Diet-induced hyperlipoproteinemia and atherosclerosis in apolipoprotein E3-Leiden transgenic mice.. J Clin Invest.

[26] NuGO microarray information page. Available: http://blog.bigcat.unimaas.nl/~martijn/NuGO/. Accessed 2008

[27] Smyth GK (2004). Linear models and empirical bayes methods for assessing differential expression in microarray experiments.. Stat Appl Genet Mol Biol.

[28] Ma P, Castillo-Davis CI, Zhong W, Liu JS (2006). A data-driven clustering method for time course gene expression data.. Nucleic Acids Res.

[29] Dennis G, Sherman BT, Hosack DA, Yang J, Gao W (2003). DAVID: Database for Annotation, Visualization, and Integrated Discovery.. Genome Biol.

[30] Subramanian A, Tamayo P, Mootha VK, Mukherjee S, Ebert BL (2005). Gene set enrichment analysis: a knowledge-based approach for interpreting genome-wide expression profiles.. Proc Natl Acad Sci U S A.

[31] Ingenuity® Systems. Available: www.ingenuity.com. Accessed 2008

[32] Koj A (1996). Initiation of acute phase response and synthesis of cytokines.. Biochim Biophys Acta.

[33] Wajant H, Pfizenmaier K, Scheurich P (2003). Tumor necrosis factor signaling.. Cell Death Differ.

[34] Gavrilova O, Haluzik M, Matsusue K, Cutson JJ, Johnson L (2003). Liver peroxisome proliferator-activated receptor gamma contributes to hepatic steatosis, triglyceride clearance, and regulation of body fat mass.. J Biol Chem.

[35] Inoue M, Ohtake T, Motomura W, Takahashi N, Hosoki Y (2005). Increased expression of PPARgamma in high fat diet-induced liver steatosis in mice.. Biochem Biophys Res Commun.

[36] Browning JD, Horton JD (2004). Molecular mediators of hepatic steatosis and liver injury.. J Clin Invest.

[37] Matsusue K, Kusakabe T, Noguchi T, Takiguchi S, Suzuki T (2008). Hepatic steatosis in leptin-deficient mice is promoted by the PPARgamma target gene Fsp27.. Cell Metab.

[38] Yu S, Matsusue K, Kashireddy P, Cao WQ, Yeldandi V (2003). Adipocyte-specific gene expression and adipogenic steatosis in the mouse liver due to peroxisome proliferator-activated receptor gamma1 (PPARgamma1) overexpression.. J Biol Chem.

[39] Luedde T, Beraza N, Kotsikoris V, van LG, Nenci A (2007). Deletion of NEMO/IKKgamma in liver parenchymal cells causes steatohepatitis and hepatocellular carcinoma.. Cancer Cell.

[40] Wunderlich FT, Luedde T, Singer S, Schmidt-Supprian M, Baumgartl J (2008). Hepatic NF-kappaB essential modulator deficiency prevents obesity-induced insulin resistance but synergizes with high-fat feeding in tumorigenesis.. Proc Natl Acad Sci U S A.

[41] Karin M (1999). The beginning of the end: IkappaB kinase (IKK) and NF-kappaB activation.. J Biol Chem.

[42] Yamaoka S, Courtois G, Bessia C, Whiteside ST, Weil R (1998). Complementation cloning of NEMO, a component of the IkappaB kinase complex essential for NF-kappaB activation.. Cell.

[43] Duque N, Gomez-Guerrero C, Egido J (1997). Interaction of IgA with Fc alpha receptors of human mesangial cells activates transcription factor nuclear factor-kappa B and induces expression and synthesis of monocyte chemoattractant protein-1, IL-8, and IFN-inducible protein 10.. J Immunol.

[44] Ouadrhiri Y, Pilette C, Monteiro RC, Vaerman JP, Sibille Y (2002). Effect of IgA on respiratory burst and cytokine release by human alveolar macrophages: role of ERK1/2 mitogen-activated protein kinases and NF-kappaB.. Am J Respir Cell Mol Biol.

[45] Drew PD, Franzoso G, Carlson LM, Biddison WE, Siebenlist U (1995). Interferon regulatory factor-2 physically interacts with NF-kappa B in vitro and inhibits NF-kappa B induction of major histocompatibility class I and beta 2-microglobulin gene expression in transfected human neuroblastoma cells.. J Neuroimmunol.

[46] Gobin SJ, Biesta P, Van den Elsen PJ (2003). Regulation of human beta 2-microglobulin transactivation in hematopoietic cells.. Blood.

[47] Poole E, Atkins E, Nakayama T, Yoshie O, Groves I (2008). NF-kappaB-mediated activation of the chemokine CCL22 by the product of the human cytomegalovirus gene UL144 escapes regulation by viral IE86.. J Virol.

[48] Pang H, Lin A, Holford M, Enerson BE, Lu B (2006). Pathway analysis using random forests classification and regression.. Bioinformatics.

[49] az-Uriarte R, varez de AS (2006). Gene selection and classification of microarray data using random forest.. BMC Bioinformatics.

[50] Uchinami H, Seki E, Brenner DA, D'Armiento J (2006). Loss of MMP 13 attenuates murine hepatic injury and fibrosis during cholestasis.. Hepatology.

[51] Read R, Hansen G, Kramer J, Finch R, Li L (2009). Ectonucleoside triphosphate diphosphohydrolase type 5 (Entpd5) deficient mice develop progressive hepatopathy, hepatocellular tumors and spermatogenic arrest.. Vet Pathol.

[52] Rodriguez JA, Buzaleh AM, Fossati M, Azcurra J, Batlle AM (2002). The effects of some porphyrinogenic drugs on the brain cholinergic system.. Cell Mol Biol (Noisy -le-grand).

[53] Iwasaki T, Yoneda M, Nakajima A, Terauchi Y (2007). Serum butyrylcholinesterase is strongly associated with adiposity, the serum lipid profile and insulin resistance.. Intern Med.

[54] Cardona F, Tunez I, Tasset I, Montilla P, Collantes E (2008). Fat overload aggravates oxidative stress in patients with the metabolic syndrome.. Eur J Clin Invest.

[55] Miyazaki M, Dobrzyn A, Sampath H, Lee SH, Man WC (2004). Reduced adiposity and liver steatosis by stearoyl-CoA desaturase deficiency are independent of peroxisome proliferator-activated receptor-alpha.. J Biol Chem.

[56] Meng F, Liu L, Chin PC, D'Mello SR (2002). Akt is a downstream target of NF-kappa B.. J Biol Chem.

[57] Ozes ON, Mayo LD, Gustin JA, Pfeffer SR, Pfeffer LM (1999). NF-kappaB activation by tumour necrosis factor requires the Akt serine-threonine kinase.. Nature.

[58] Romashkova JA, Makarov SS (1999). NF-kappaB is a target of AKT in anti-apoptotic PDGF signalling.. Nature.

[59] Manning BD, Cantley LC (2007). AKT/PKB signaling: navigating downstream.. Cell.

[60] Spiegelman BM, Flier JS (1996). Adipogenesis and obesity: rounding out the big picture.. Cell.

[61] Kim JB, Spiegelman BM (1996). ADD1/SREBP1 promotes adipocyte differentiation and gene expression linked to fatty acid metabolism.. Genes Dev.

[62] Suzawa M, Takada I, Yanagisawa J, Ohtake F, Ogawa S (2003). Cytokines suppress adipogenesis and PPAR-gamma function through the TAK1/TAB1/NIK cascade.. Nat Cell Biol.

[63] Ricote M, Glass CK (2007). PPARs and molecular mechanisms of transrepression.. Biochim Biophys Acta.

[64] Zingarelli B, Sheehan M, Hake PW, O'Connor M, Denenberg A (2003). Peroxisome proliferator activator receptor-gamma ligands, 15-deoxy-Delta(12,14)-prostaglandin J2 and ciglitazone, reduce systemic inflammation in polymicrobial sepsis by modulation of signal transduction pathways.. J Immunol.

[65] Straus DS, Pascual G, Li M, Welch JS, Ricote M (2000). 15-deoxy-delta 12,14-prostaglandin J2 inhibits multiple steps in the NF-kappa B signaling pathway.. Proc Natl Acad Sci U S A.

[66] Rossi A, Kapahi P, Natoli G, Takahashi T, Chen Y (2000). Anti-inflammatory cyclopentenone prostaglandins are direct inhibitors of IkappaB kinase.. Nature.

[67] Wellen KE, Hotamisligil GS (2005). Inflammation, stress, and diabetes.. J Clin Invest.

[68] Cai D, Yuan M, Frantz DF, Melendez PA, Hansen L (2005). Local and systemic insulin resistance resulting from hepatic activation of IKK-beta and NF-kappaB.. Nat Med.

[69] Shoelson SE, Lee J, Goldfine AB (2006). Inflammation and insulin resistance.. J Clin Invest.

[70] Li AC, Brown KK, Silvestre MJ, Willson TM, Palinski W (2000). Peroxisome proliferator-activated receptor gamma ligands inhibit development of atherosclerosis in LDL receptor-deficient mice.. J Clin Invest.

[71] Collins AR, Meehan WP, Kintscher U, Jackson S, Wakino S (2001). Troglitazone inhibits formation of early atherosclerotic lesions in diabetic and nondiabetic low density lipoprotein receptor-deficient mice.. Arterioscler Thromb Vasc Biol.

[72] Haffner SM, Greenberg AS, Weston WM, Chen H, Williams K (2002). Effect of rosiglitazone treatment on nontraditional markers of cardiovascular disease in patients with type 2 diabetes mellitus.. Circulation.

[73] Kuenzli S, Saurat JH (2003). Peroxisome proliferator-activated receptors in cutaneous biology.. Br J Dermatol.

[74] Su CG, Wen X, Bailey ST, Jiang W, Rangwala SM (1999). A novel therapy for colitis utilizing PPAR-gamma ligands to inhibit the epithelial inflammatory response.. J Clin Invest.

[75] ArrayExpress repository. Available: www.ebi.ac.uk/arrayexpress/. Accessed 2008

[76] The R Project for Statistical Computing. Available: http://www.r-project.org/. Accessed 2008

[77] Bioconductor. Available: http://www.bioconductor.org/. Accessed 2008

[78] Management and Analysis Database for MicroArray eXperiments (MADMAX). Available: https://madmax.bioinformatics.nl. Accessed 2008

[79] Molecular and Behavioral Neuroscience Institute. Available: http://brainarray.mbni.med.umich.edu/. Accessed 2008

[80] Dai M, Wang P, Boyd AD, Kostov G, Athey B (2005). Evolving gene/transcript definitions significantly alter the interpretation of GeneChip data.. Nucleic Acids Res.

[81] Reich M, Liefeld T, Gould J, Lerner J, Tamayo P (2006). GenePattern 2.0.. Nat Genet.

[82] Eisen MB, Spellman PT, Brown PO, Botstein D (1998). Cluster analysis and display of genome-wide expression patterns.. Proc Natl Acad Sci U S A.

[83] de Hoon MJ, Imoto S, Nolan J, Miyano S (2004). Open source clustering software.. Bioinformatics.

[84] Liaw A, Wiener M (2002). Classification and Regression by randomForest.. R News.

