# Supplementary material for: Genome-Wide mRNA Expression Analysis of Hepatic Adaptation to High-Fat Diets Reveals Switch from an Inflammatory to Steatotic Transcriptional Program
Source: PLoS One. 2009 Aug 14;4(8):e6646. doi: 10.1371/journal.pone.0006646 (PMC2722023; doi:10.1371/journal.pone.0006646)

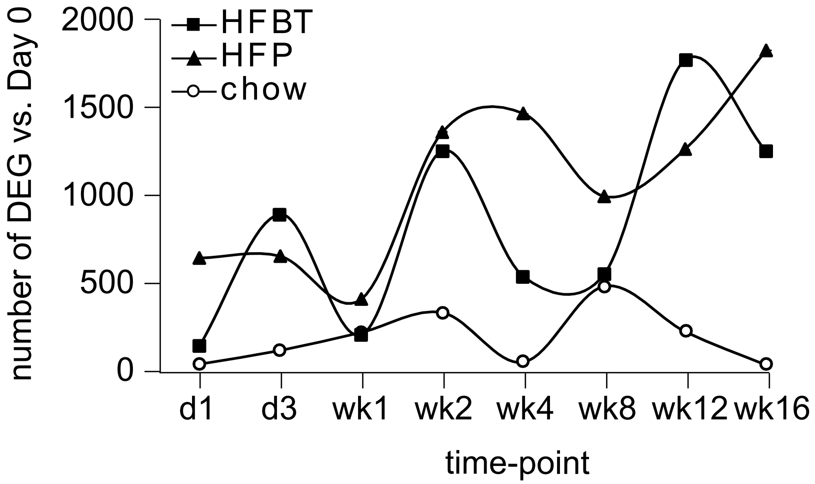

	HFBT vs. chow Day 0	HFP vs. chow Day 0	chow vs. chow Day0
Day 1	110	626	6
Day 3	886	637	86
Week 1	176	383	191
Week 2	1260	1371	306
Week 4	520	1482	18
Week 8	534	991	460
Week12	1796	1271	195
Week 16	1258	1855	1
Total number of unique genes	3027	3316	839

Overlap of significant genes (total) in each of 3 diets

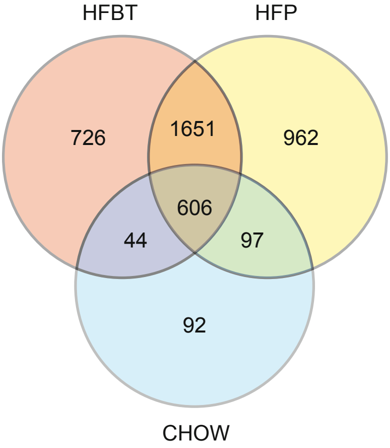

Supplement: Figure S1 — Temporal changes in gene expression for HFBT, HFP and chow diet over the 16-week period compared to day 0. The number of statistically significant differentially expressed genes (DEG) identified by pairwise comparison of each time-point versus day 0 (limma, FDR<0.1) in chow, HFBT and HFP dietary conditions. Venn diagram shows overlap of total (in any of time points) number of DEGs in the three diets. (0.37 MB PDF) [file pone.0006646.s001.pdf]
