# Supplementary figures and images for: Genome-Wide mRNA Expression Analysis of Hepatic Adaptation to High-Fat Diets Reveals Switch from an Inflammatory to Steatotic Transcriptional Program
Source: PLoS One. 2009 Aug 14;4(8):e6646. doi: 10.1371/journal.pone.0006646 (PMC2722023; doi:10.1371/journal.pone.0006646)

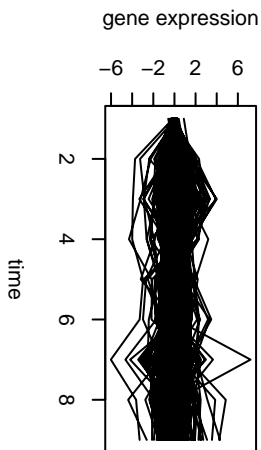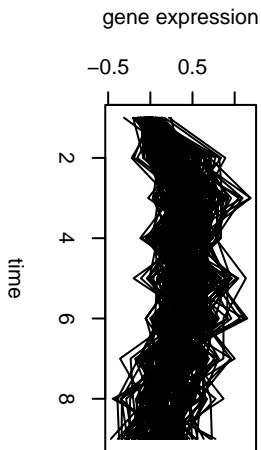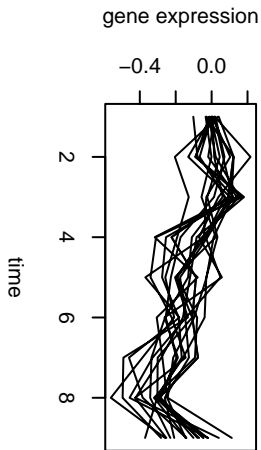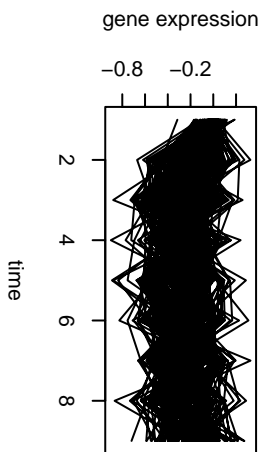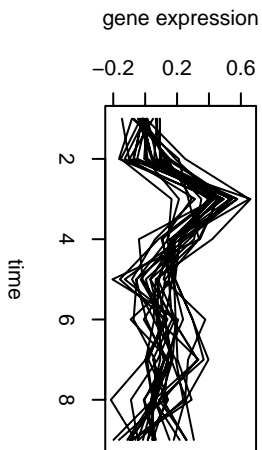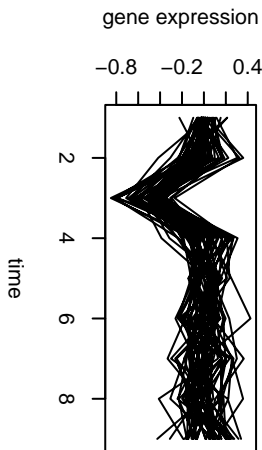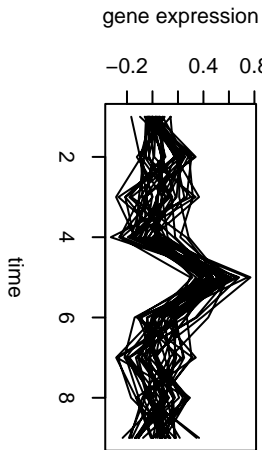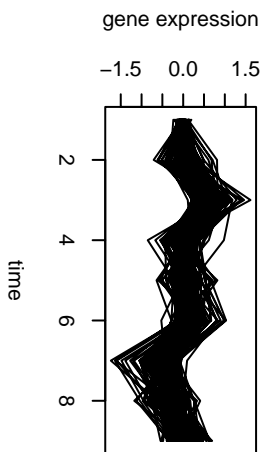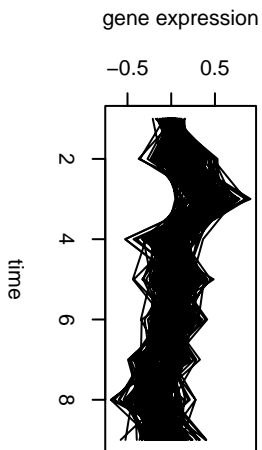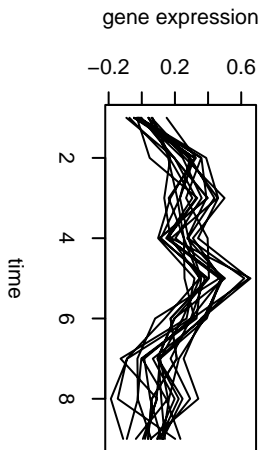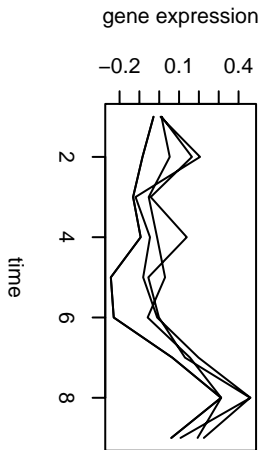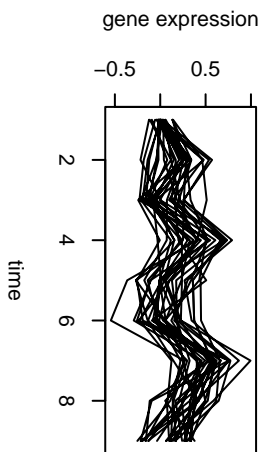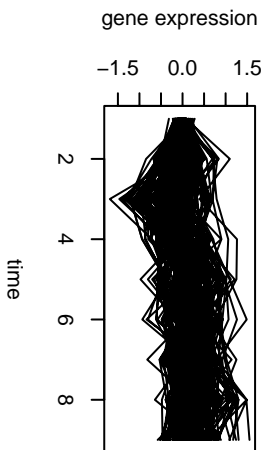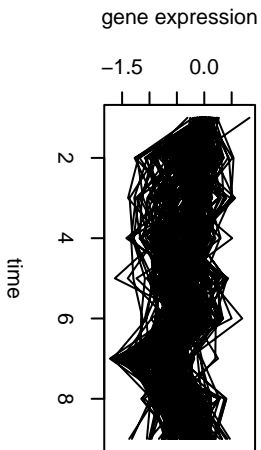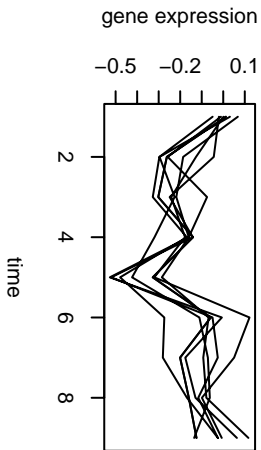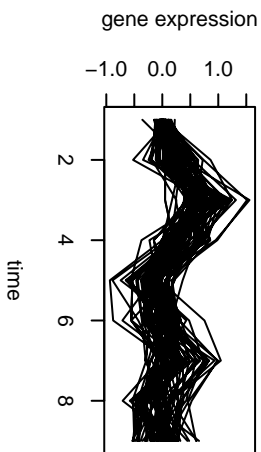

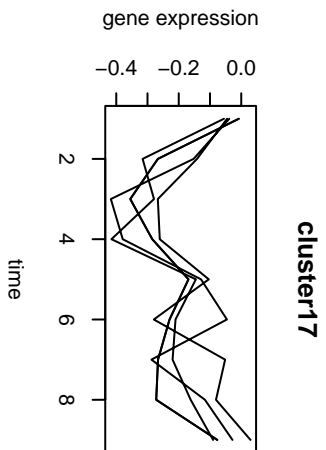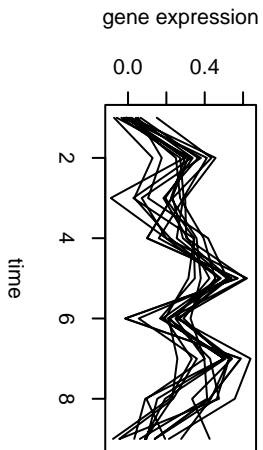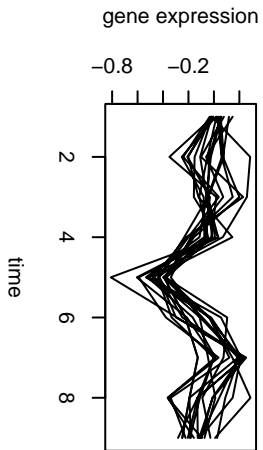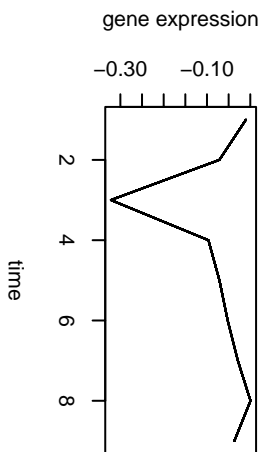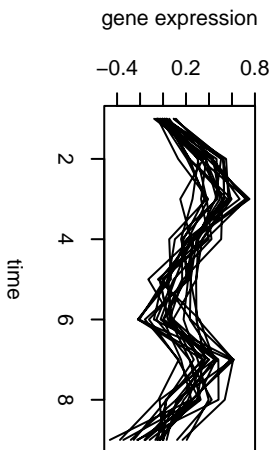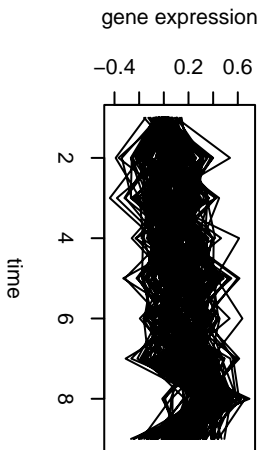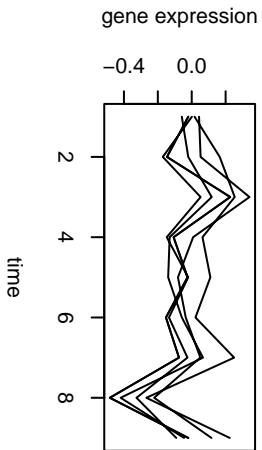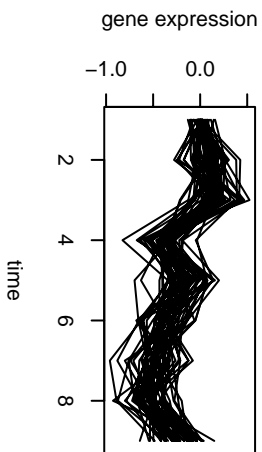

Supplement: Figure S2 — Temporal gene expression profiles (HFBT, raw curves). The results of Smoothing Spline Clustering analysis [25] for 1663 high-fat responsive genes. The genes are grouped into 24 clusters according to their temporal expression profiles. The vertical axis represents the expression ratios and the horizontal axis the time points 1 to 9 (day 0, day 1, day 3, week 1, week 2, week 4, week 8, week 12 and week 16). Figure S2 corresponds to the HFBT experimental conditions and raw expression ratio values. (0.07 MB PDF) [file pone.0006646.s002.pdf]

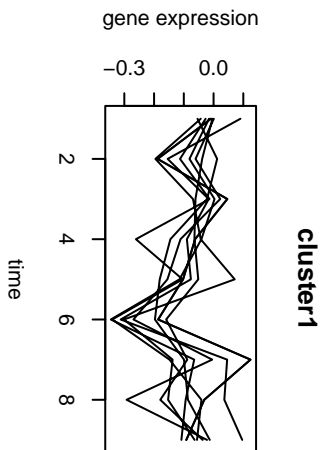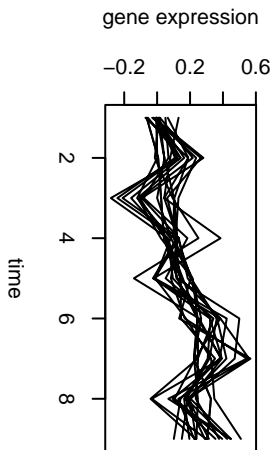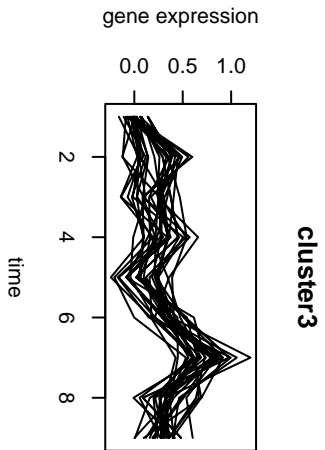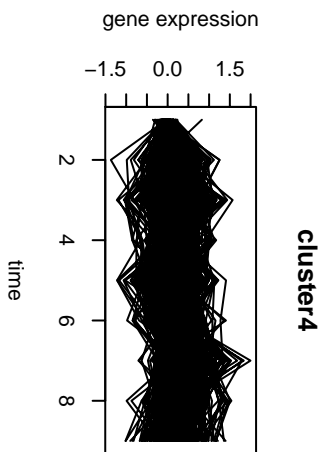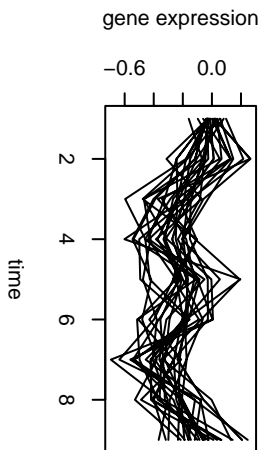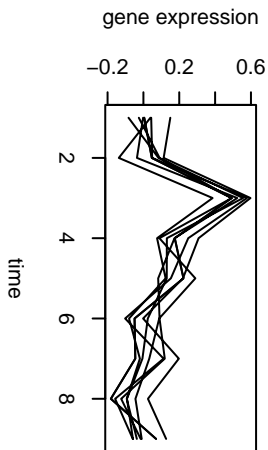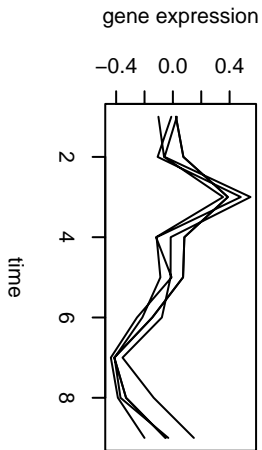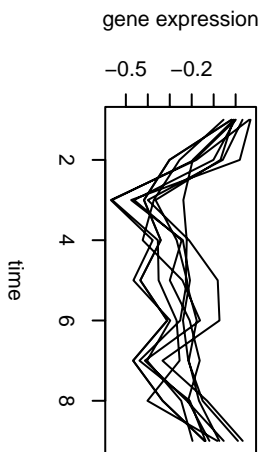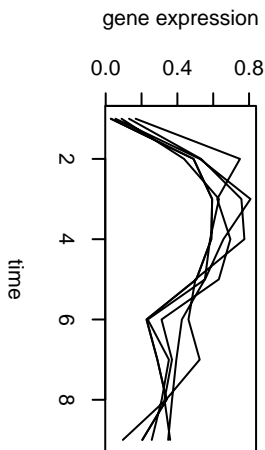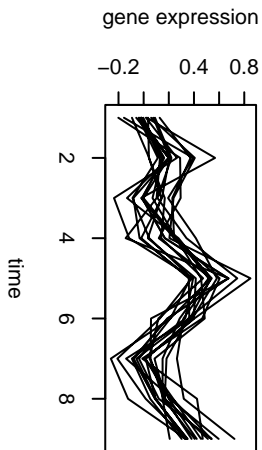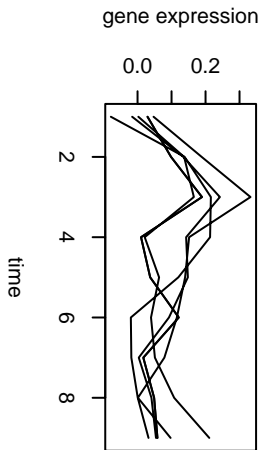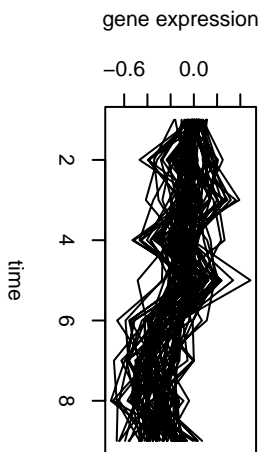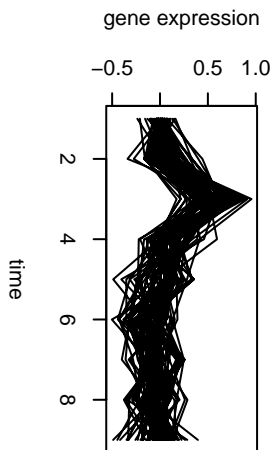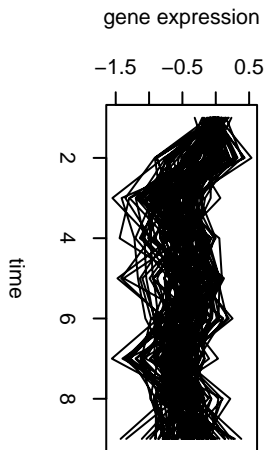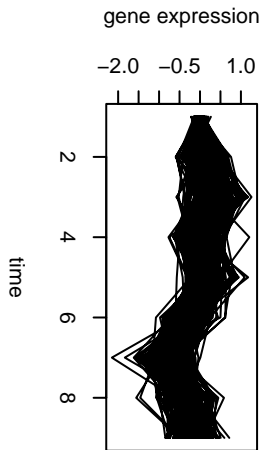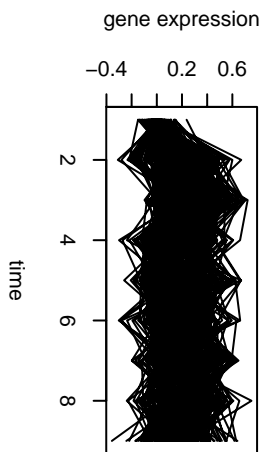

**cluster17**

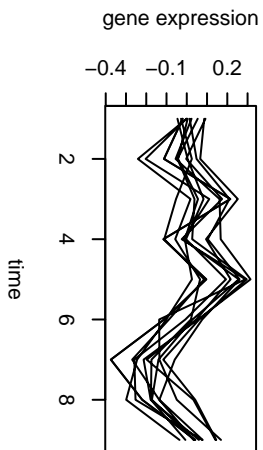

**cluster18**

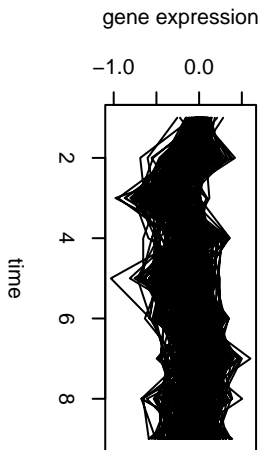

**cluster19**

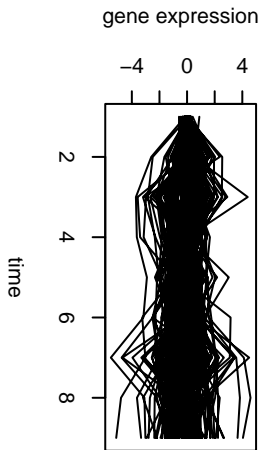

**cluster20**

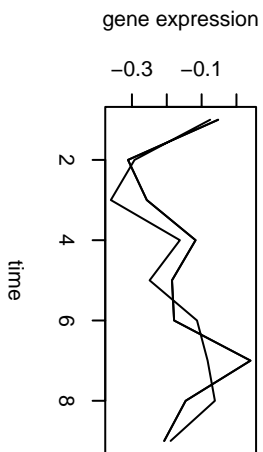

**cluster21**

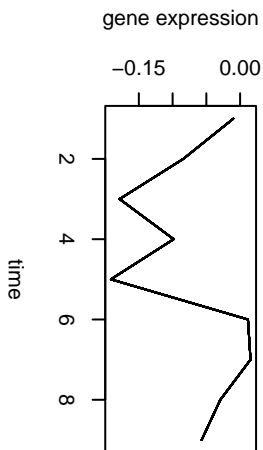

**cluster22**

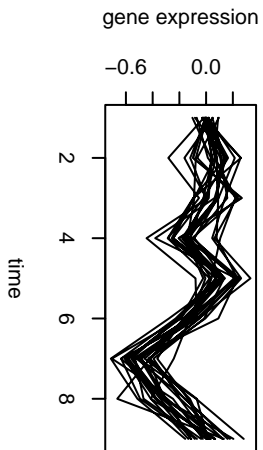

**cluster23**

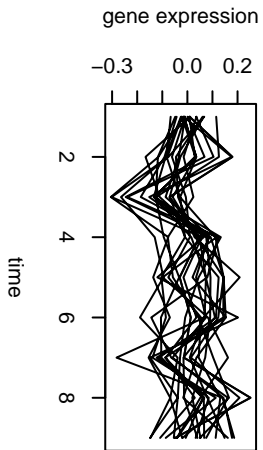

**cluster24**

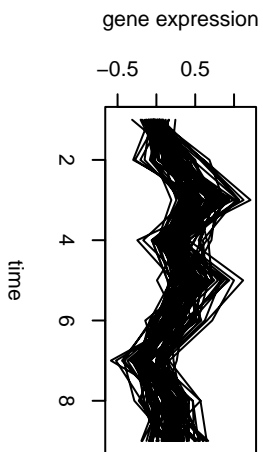

Supplement: Figure S4 — Temporal gene expression profiles (HFP, raw curves). The results of Smoothing Spline Clustering analysis [25] for 1663 high-fat responsive genes. The genes are grouped into 24 clusters according to their temporal expression profiles. The vertical axis represents the expression ratios and the horizontal axis the time points 1 to 9 (day 0, day 1, day 3, week 1, week 2, week 4, week 8, week 12 and week 16). Figure S4 corresponds to the HFP experimental conditions and raw expression ratio values. (0.07 MB PDF) [file pone.0006646.s004.pdf]
