# Supplementary material for: Genome-Wide mRNA Expression Analysis of Hepatic Adaptation to High-Fat Diets Reveals Switch from an Inflammatory to Steatotic Transcriptional Program
Source: PLoS One. 2009 Aug 14;4(8):e6646. doi: 10.1371/journal.pone.0006646 (PMC2722023; doi:10.1371/journal.pone.0006646)
Supplement: Figure S3 — Temporal gene expression profiles (HFBT, mean curves). The results of Smoothing Spline Clustering analysis [25] for 1663 high-fat responsive genes. The genes are grouped into 24 clusters according to their temporal expression profiles. The vertical axis represents the expression ratios and the horizontal axis the time points 1 to 9 (day 0, day 1, day 3, week 1, week 2, week 4, week 8, week 12 and week 16). Figure S3 corresponds to the HFBT experimental conditions and mean expression ratio values (and their confidence intervals, in red) of the genes in each cluster. (0.02 MB PDF) [file pone.0006646.s003.pdf]

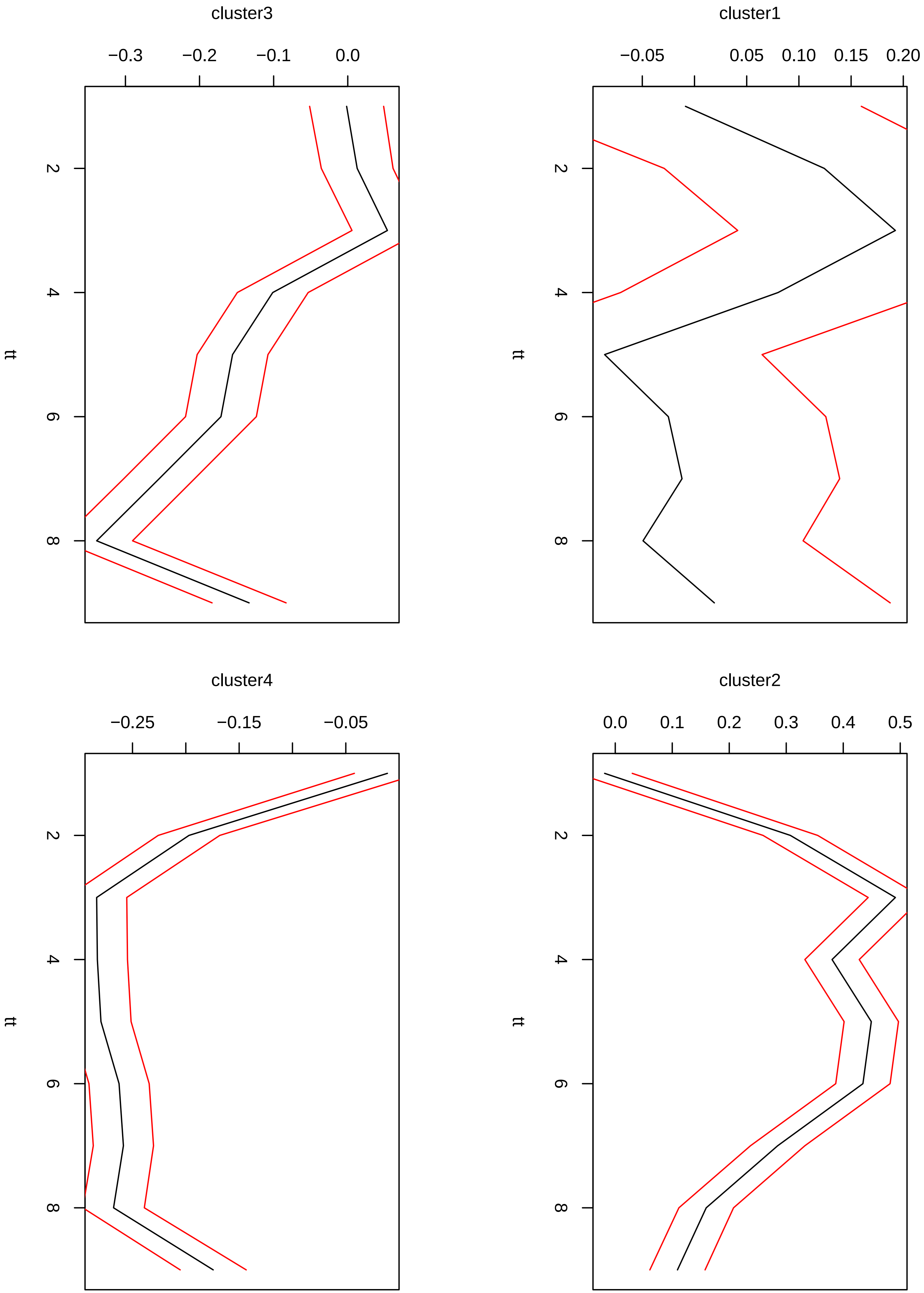
cluster3
−0.3
−0.2
−0.1
0.0
tt
2
4
6
8
cluster1
−0.05
0.05
0.10
0.15
0.20
tt
2
4
6
8
cluster4
−0.25
−0.15
−0.05
tt
2
4
6
8
cluster2
0.0
0.1
0.2
0.3
0.4
0.5
tt
2
4
6
8

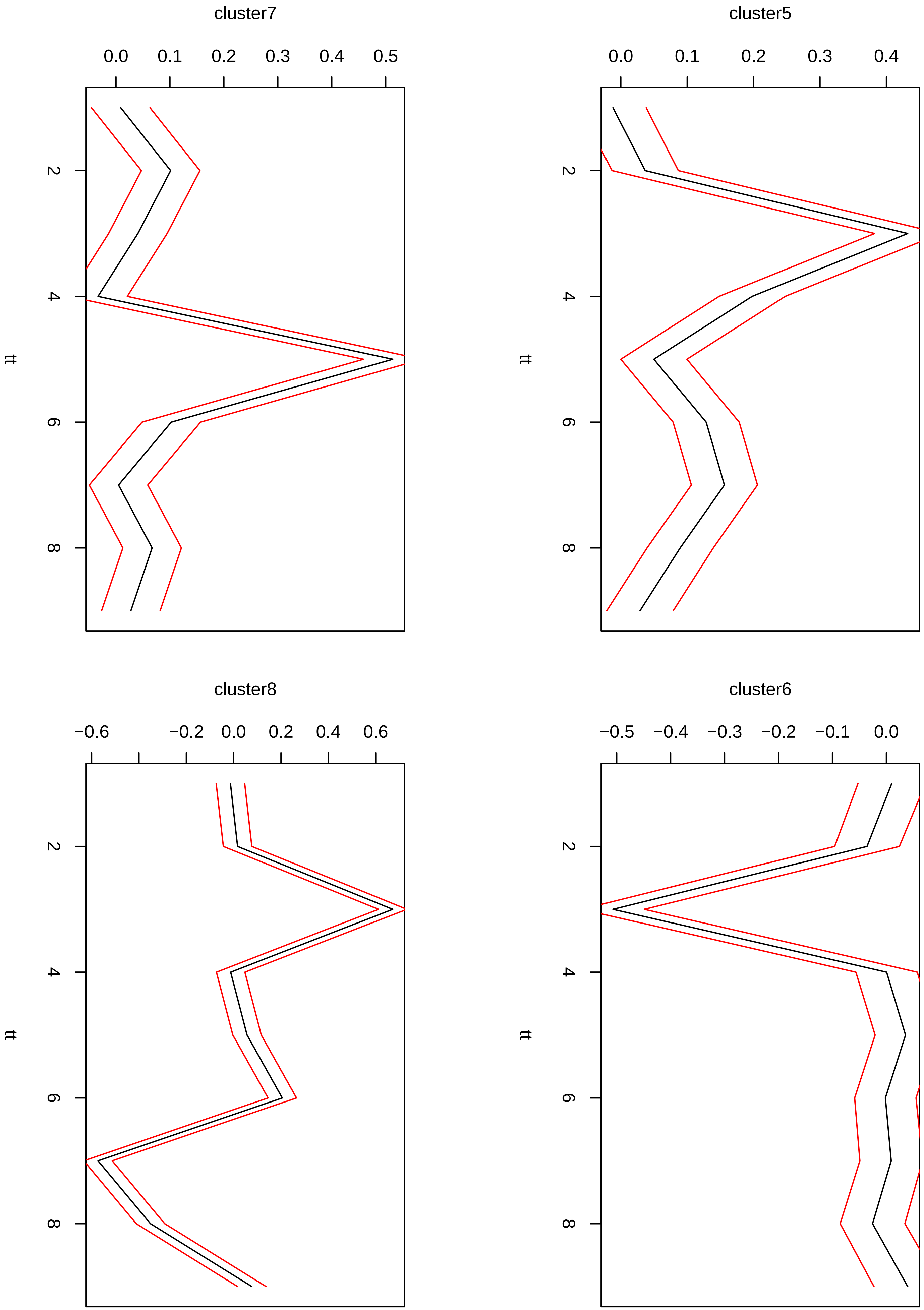
cluster7
0.0
0.1
0.2
0.3
0.4
0.5
2
4
6
8
tt
cluster5
0.0
0.1
0.2
0.3
0.4
2
4
6
8
tt
cluster8
−0.6
−0.2
0.0
0.2
0.4
0.6
2
4
6
8
tt
cluster6
−0.5
−0.4
−0.3
−0.2
−0.1
0.0
2
4
6
8
tt

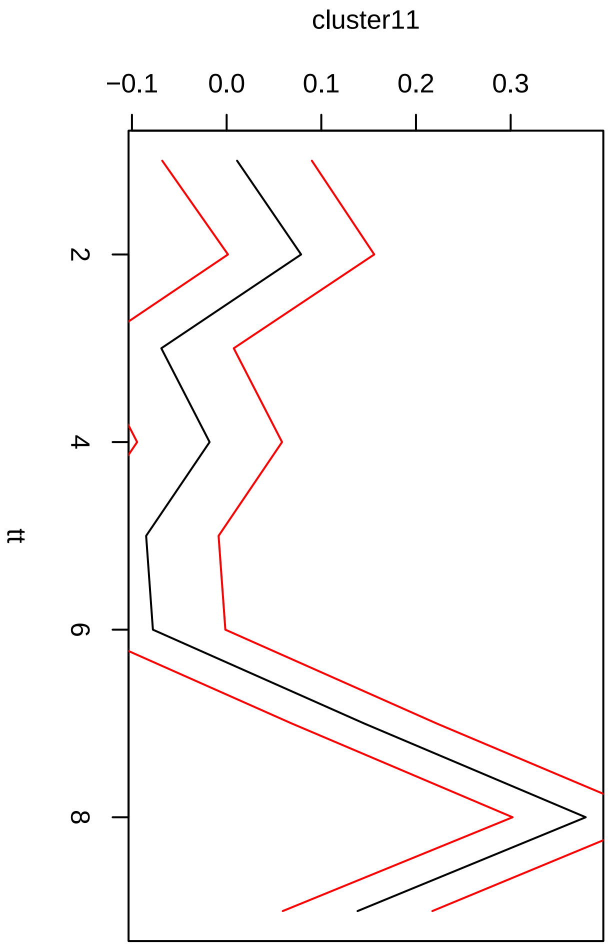

cluster11
−0.1
0.0
0.1
0.2
0.3
tt
2
4
6
8

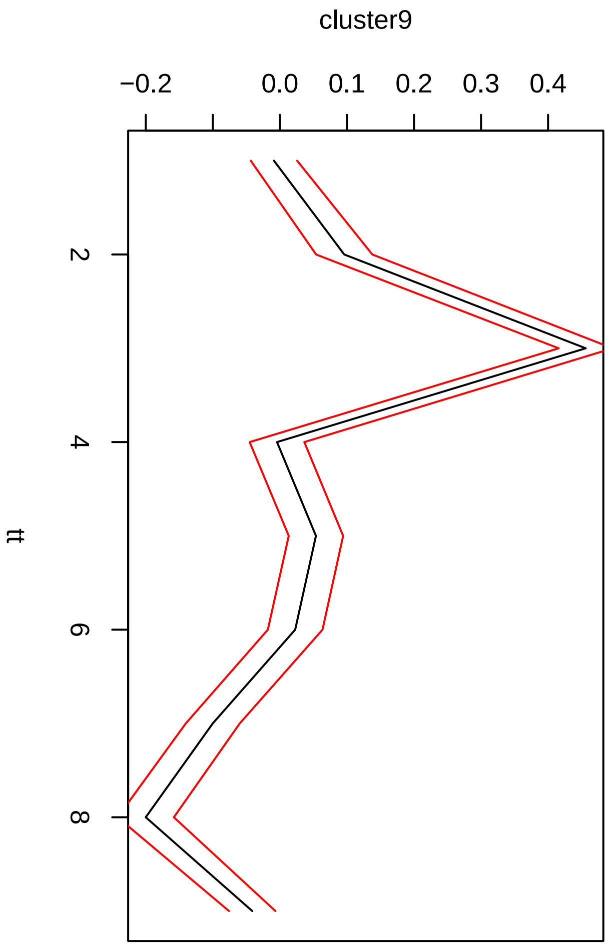

cluster9
−0.2
0.0
0.1
0.2
0.3
0.4
tt
2
4
6
8

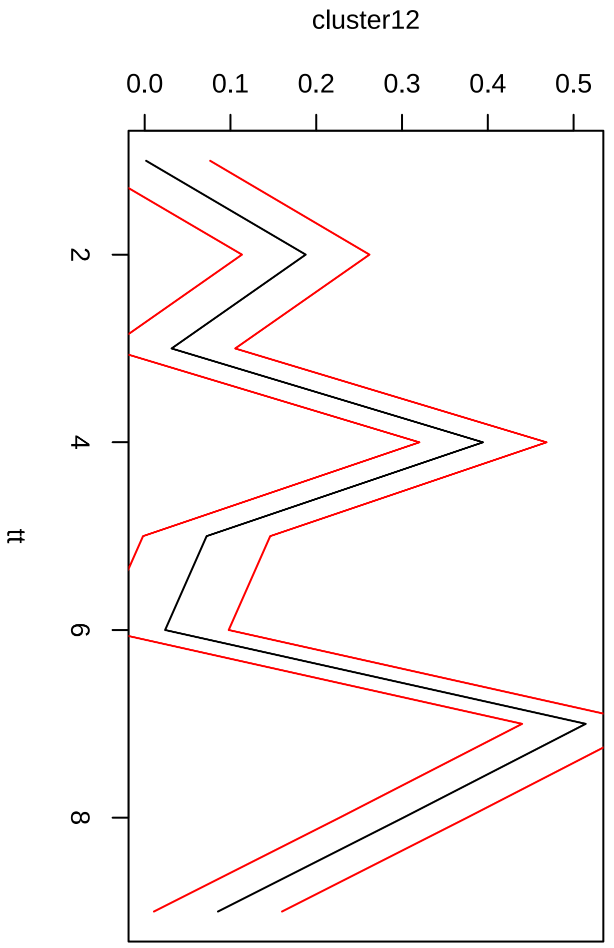

cluster12
0.0
0.1
0.2
0.3
0.4
0.5
tt
2
4
6
8

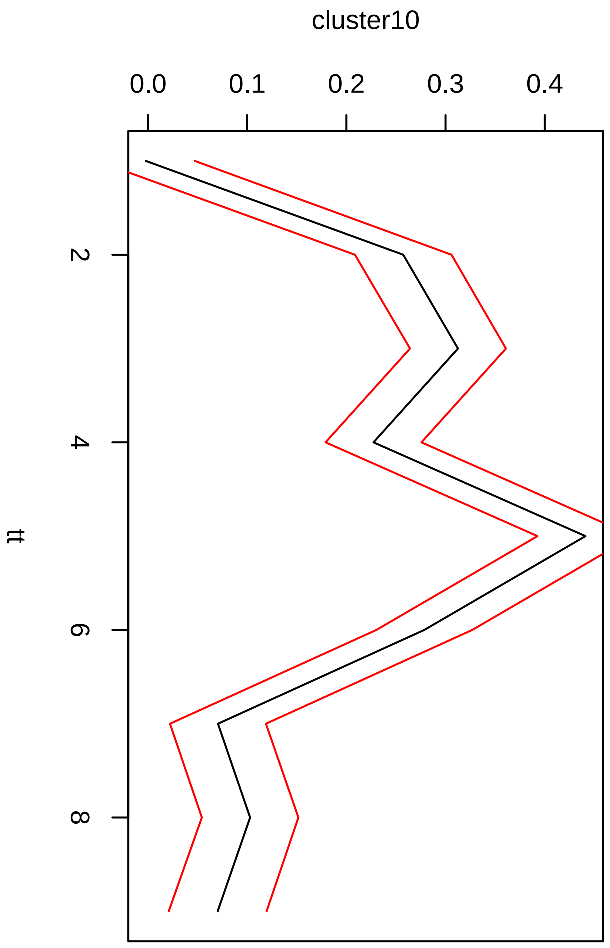

cluster10
0.0
0.1
0.2
0.3
0.4
tt
2
4
6
8

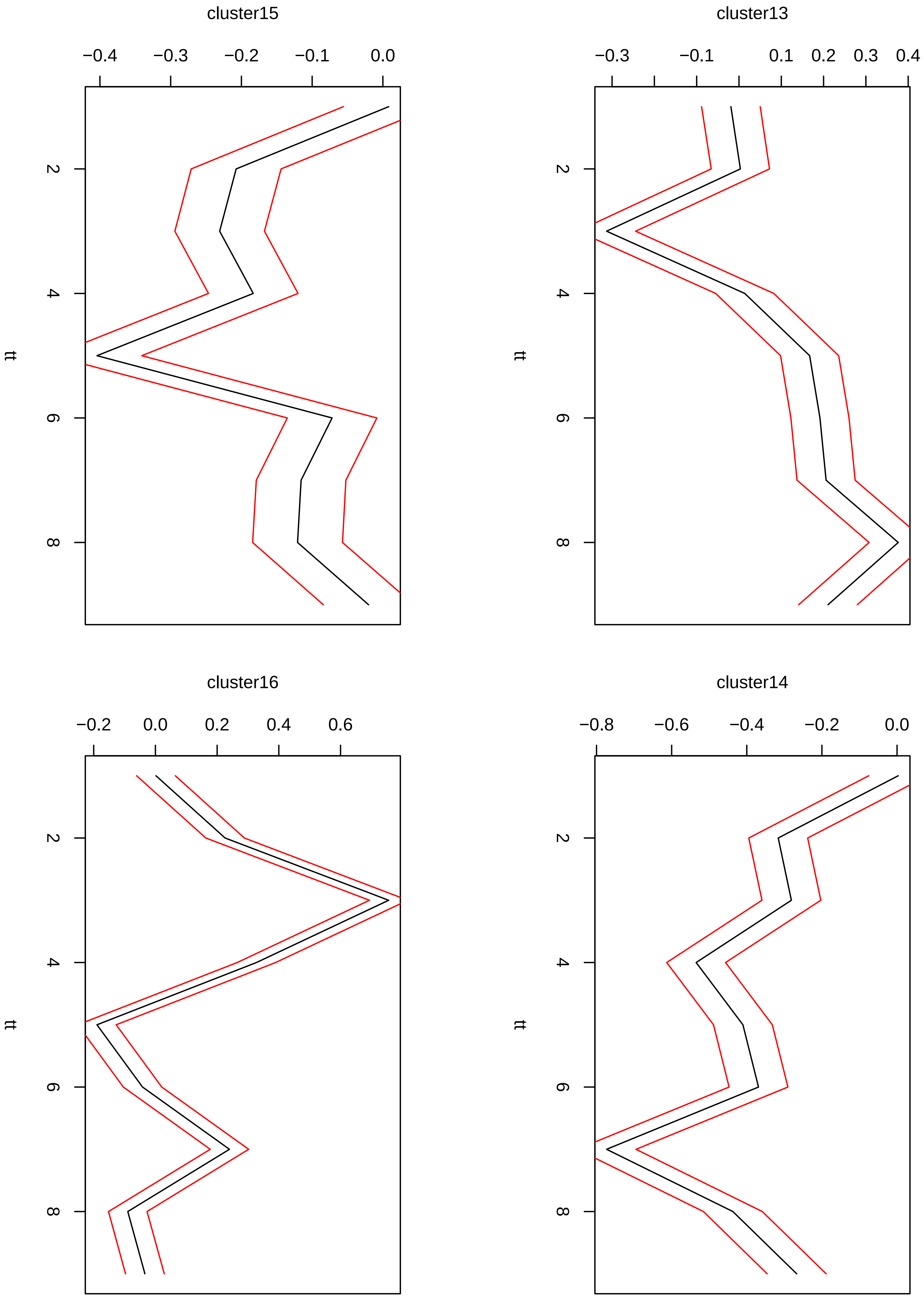
cluster15
cluster13
cluster16
cluster14
tt
−0.4
−0.3
−0.2
−0.1
0.0
0.1
0.2
0.3
0.4
−0.8
−0.6
0.6
2
4
6
8

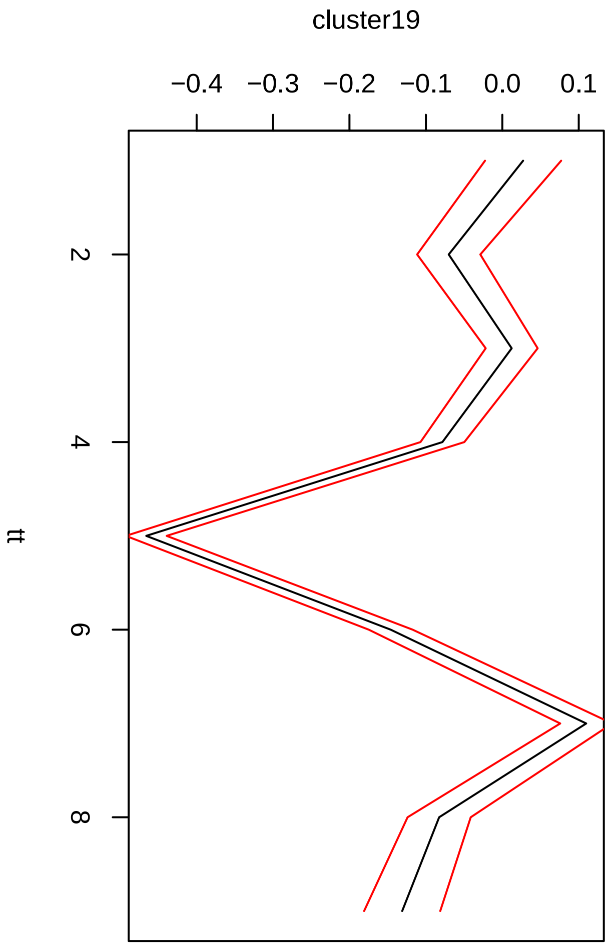
cluster19
−0.4
−0.3
−0.2
−0.1
0.0
0.1
2
4
6
8
tt
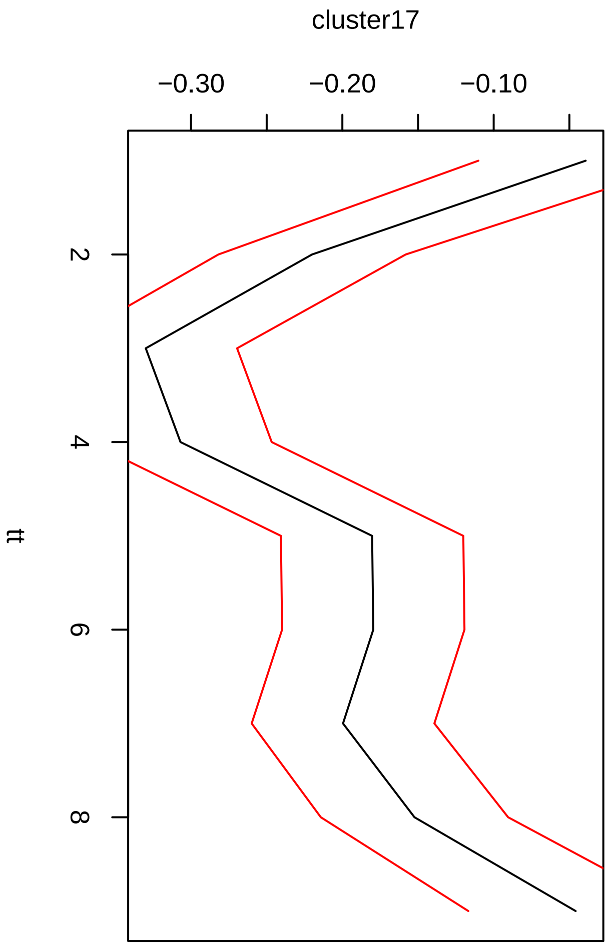
cluster17
−0.30
−0.20
−0.10
2
4
6
8
tt
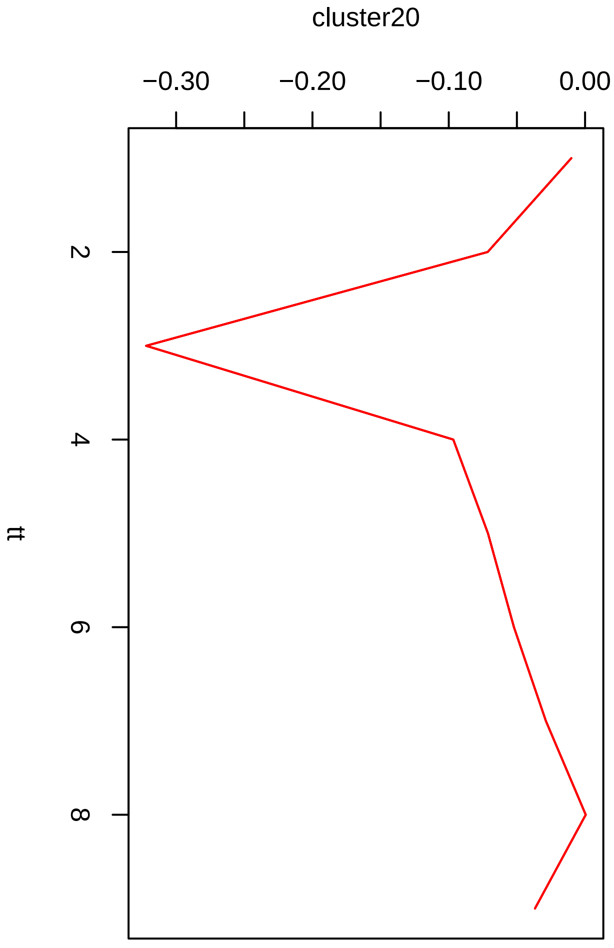
cluster20
−0.30
−0.20
−0.10
0.00
2
4
6
8
tt
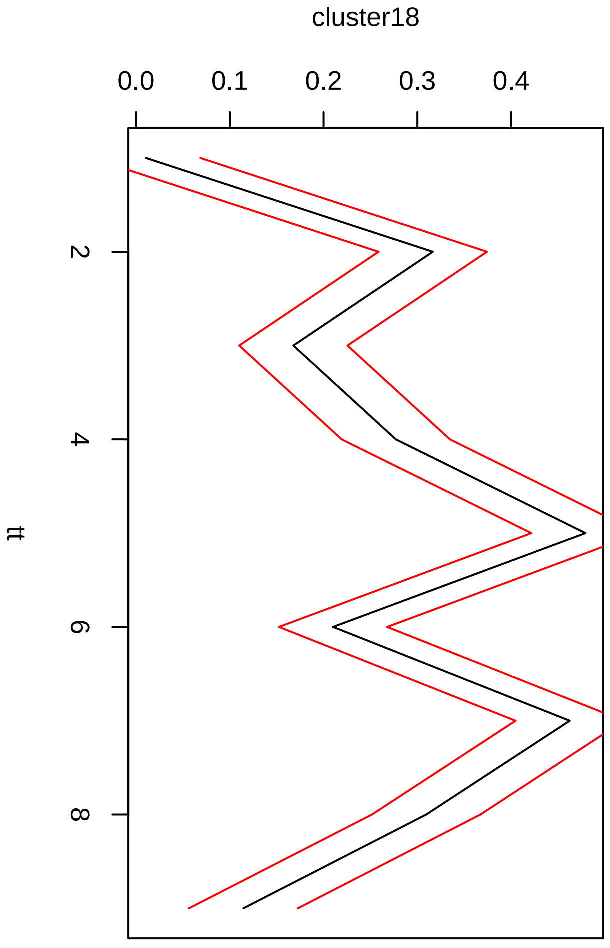
cluster18
0.0
0.1
0.2
0.3
0.4
2
4
6
8
tt

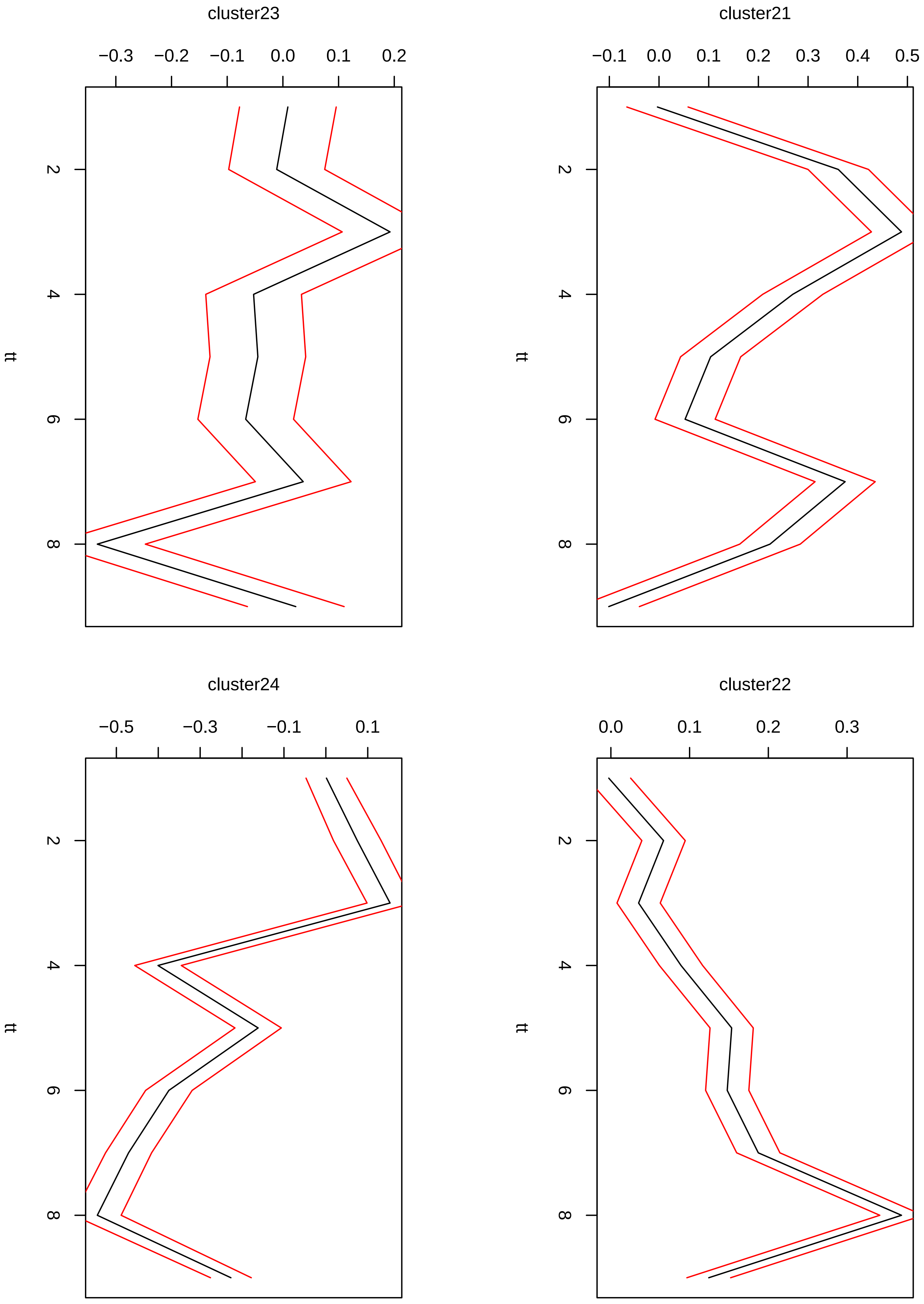
cluster23
cluster21
cluster24
cluster22
tt
−0.3 −0.2 −0.1 0.0 0.1 0.2
−0.1 0.0 0.1 0.2 0.3 0.4 0.5
−0.5 −0.3 −0.1 0.1
0.0 0.1 0.2 0.3
2 4 6 8
