# Supplementary material for: Genome-Wide mRNA Expression Analysis of Hepatic Adaptation to High-Fat Diets Reveals Switch from an Inflammatory to Steatotic Transcriptional Program
Source: PLoS One. 2009 Aug 14;4(8):e6646. doi: 10.1371/journal.pone.0006646 (PMC2722023; doi:10.1371/journal.pone.0006646)
Supplement: Table S1 — Detailed overview of numbers of differentially expressed genes in HFBT vs. chow and HFP vs. chow comparisons (limma, FDR<0.1). The information summarized in Table 1 is extended to a detail to include: (1) number of upregulated genes per diet and time point, (2) number of downregulated genes per diet and time point, (3) total (both up- and downregulated) number of differentially expressed genes per diet and time point, (4) number of overlapping genes between HFBT and HFP diets for each of categories (1)–(3), (5) number of genes in union of HFBT and HFP diets for each of categories (1)–(3) and (6) total (in all time-points) number of differentially expressed genes for categories (1)–(5). (0.05 MB DOC) [file pone.0006646.s006.doc]

| Time point | HFBT vs. chow | | | HFP vs. chow | | | Overlap  (HFBT vs. chow)  and  (HFP vs. chow) | | | Union  (HFBT vs. chow)  or  (HFP vs. chow) | | |
| --- | --- | --- | --- | --- | --- | --- | --- | --- | --- | --- | --- | --- |
|  | Up | Down | Total | Up | Down | Total | Up | Down | Total | Up | Down | Total |
| Day 1 | 0 | 0 | 0 | 0 | 0 | 0 | 0 | 0 | 0 | 0 | 0 | 0 |
| Day 3 | 345 | 205 | 550 | 265 | 151 | 416 | 125 | 68 | 193 | 485 | 288 | 773 |
| Week 1 | 26 | 62 | 88 | 32 | 50 | 82 | 5 | 26 | 31 | 53 | 86 | 139 |
| Week 2 | 178 | 139 | 317 | 77 | 59 | 136 | 45 | 31 | 76 | 210 | 167 | 377 |
| Week 4 | 18 | 15 | 33 | 17 | 27 | 44 | 4 | 5 | 9 | 31 | 37 | 68 |
| Week 8 | 3 | 4 | 7 | 74 | 135 | 209 | 0 | 2 | 2 | 77 | 137 | 214 |
| Week 12 | 239 | 282 | 521 | 51 | 93 | 144 | 23 | 62 | 85 | 267 | 313 | 580 |
| Week 16 | 16 | 7 | 23 | 22 | 30 | 52 | 6 | 1 | 7 | 32 | 36 | 68 |
| All time-points | **702** | **579** | **1263** | **441** | **401** | **836** | **221** | **194** | **436** | **922** | **786** | **1663** |
