# Supplementary material for: Genome-Wide mRNA Expression Analysis of Hepatic Adaptation to High-Fat Diets Reveals Switch from an Inflammatory to Steatotic Transcriptional Program
Source: PLoS One. 2009 Aug 14;4(8):e6646. doi: 10.1371/journal.pone.0006646 (PMC2722023; doi:10.1371/journal.pone.0006646)
Supplement: Table S4 — The macronutrient content and the fatty acid composition of chow and high-fat diets. The macronutrient content and the fatty acid composition of chow, HFBT and HFP diets. (0.04 MB DOC) [file pone.0006646.s009.doc]

| **Macronutrients** | **Chow** | **HFBT** | **HFP** |
| --- | --- | --- | --- |
| Protein (%) | 20.0 | 24.0 | 23.3 |
| Carbohydrate (%) | 63.0 | 39.0 | 40.2 |
| Fat (%) | 7.0 | 24.3 | 23.6 |
| Fiber (%) | 5.0 | 6.0 | 5.8 |
| Other (%) | 5.0 | 6.7 | 7.0 |
| Total | 100 | 100 | 99.9 |
| **Fatty acids (g/100g fat)** | | | |
| C12:0 | - | - | 0.3 |
| C14:0 | - | 0.8 | 0.9 |
| C16:0 | 10.0 | 28.9 | 35.4 |
| C16:1w7 | - | 2.8 | 0.2 |
| C18:0 | 3.8 | 14.8 | 4.4 |
| C18:1w9 | 21.0 | 42.9 | 40.4 |
| C18:2w6 | 52.0 | 8.8 | 16.4 |
| C18:3w3 | 4.8 | - | 0.7 |
| C20:0 | 0.5 | - | 0.5 |
| C20:1w9 | 0.2 | - | 0.1 |
| C22:0 | 0.5 | - | 0.2 |
| cholesterol | - | 1.0 | - |
